# β-Amino Acids Reduce Ternary Complex Stability and Alter the Translation Elongation Mechanism

**DOI:** 10.1021/acscentsci.4c00314

**Published:** 2024-06-04

**Authors:** F. Aaron Cruz-Navarrete, Wezley C. Griffin, Yuk-Cheung Chan, Maxwell I. Martin, Jose L. Alejo, Ryan A. Brady, S. Kundhavai Natchiar, Isaac J. Knudson, Roger B. Altman, Alanna Schepartz, Scott J. Miller, Scott C. Blanchard

**Affiliations:** † Department of Structural Biology, St. Jude Children’s Research Hospital, Memphis, Tennessee 38105, United States; ‡ Department of Chemical Biology & Therapeutics, St. Jude Children’s Research Hospital, Memphis, Tennessee 38105, United States; § Department of Chemistry, Yale University, New Haven, Connecticut 06511, United States; ∥ College of Chemistry, University of California, Berkeley, Berkeley, California 94720, United States; ⊥ Molecular and Cell Biology, University of California, Berkeley, Berkeley, California 94720, United States; # California Institute for Quantitative Biosciences, University of California, Berkeley, Berkeley, California 94720, United States; ∇ Chan Zuckerberg Biohub, San Francisco, California 94158, United States; ○ Innovation Investigator, ARC Institute, Palo Alto, California 94304, United States

## Abstract

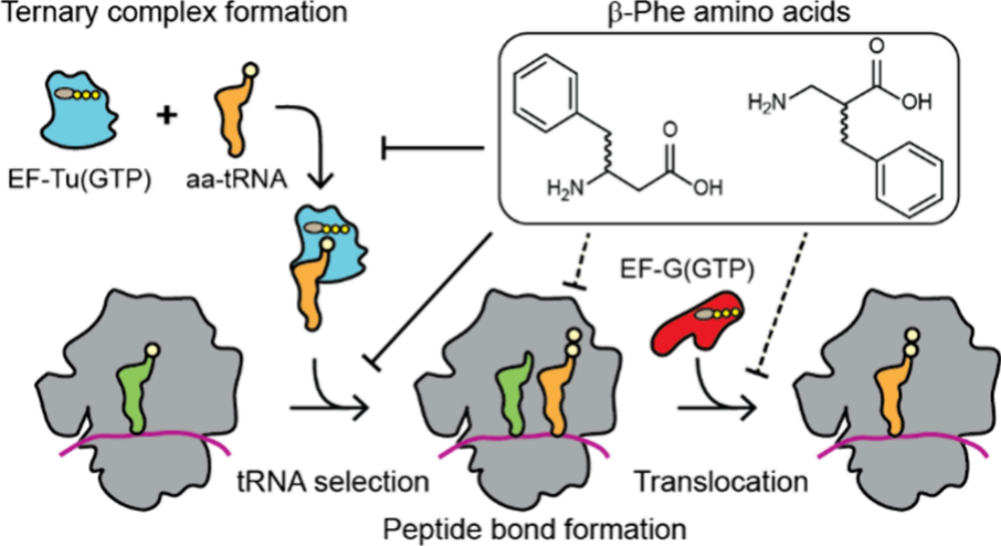

Templated synthesis of proteins containing non-natural amino acids (nnAAs) promises to expand the chemical space available to biological therapeutics and materials, but existing technologies are still limiting. Addressing these limitations requires a deeper understanding of the mechanism of protein synthesis and how it is perturbed by nnAAs. Here we examine the impact of nnAAs on the formation and ribosome utilization of the central elongation substrate: the ternary complex of native, aminoacylated tRNA, thermally unstable elongation factor, and GTP. By performing ensemble and single-molecule fluorescence resonance energy transfer measurements, we reveal that both the (*R*)- and (*S*)-β^2^ isomers of phenylalanine (Phe) disrupt ternary complex formation to levels below in vitro detection limits, while (*R*)- and (*S*)-β^3^-Phe reduce ternary complex stability by 1 order of magnitude. Consistent with these findings, (*R*)- and (*S*)-β^2^-Phe-charged tRNAs were not utilized by the ribosome, while (*R*)- and (*S*)-β^3^-Phe stereoisomers were utilized inefficiently. (*R*)-β^3^-Phe but not (*S*)-β^3^-Phe also exhibited order of magnitude defects in the rate of translocation after mRNA decoding. We conclude from these findings that non-natural amino acids can negatively impact the translation mechanism on multiple fronts and that the bottlenecks for improvement must include the consideration of the efficiency and stability of ternary complex formation.

## Abstract 2

β-Phe amino acids perturb several critical steps of the protein synthesis mechanism, revealing previously unappreciated bottlenecks that limit noncanonical amino acid incorporation into proteins.

## Introduction

The mechanism of ribosome-catalyzed polypeptide polymerization offers the opportunity to perform template-driven synthesis of nonprotein polymers with benefits for both fundamental research and biomedicine. Over the last several decades, substantial progress has been made toward expanding the genetic code to increase the expressed proteome well beyond the 20 natural α-amino acids. More than 200 different non-natural α-amino acids (nnAAs) and a handful of α-hydroxy acids can be incorporated into proteins.^1−5^ There are now several examples of β^2^- and/or β^3^-monomers that have been introduced into proteins in cells, either directly^6−8^ or via rearrangement.^9^ Robust methods of α- and non-α nnAA incorporation into proteins are expected to promote the development of new tools to probe structure–function relationships, discover catalysts, and advance therapeutic approaches.

The most widely adopted nnAA incorporation strategies require the transplantation of translation components from orthogonal biological systems into model organisms (e.g., *Escherichia coli*) to promote the selective formation of the desired aminoacyl-tRNA (aa-tRNA).^10,11^ Inefficient suppressor tRNA aminoacylation (charging) initially represented a severe limitation, which was largely overcome through the engineering of native aminoacyl-tRNA synthases (aaRS).^12,13^ Such strides have permitted the incorporation of α-amino acids with distinct non-natural side chains into otherwise native proteins.^11,14^ However, the translational machinery did not evolve to support the translation of components with alternative backbones, and significant evidence exists that shows multiple kinetic bottlenecks likely exist.^7,15,16^ Here we identify bottlenecks that exist during the delivery and accommodation of nnAA-acylated tRNA by the ribosome.

**Figure 1:**
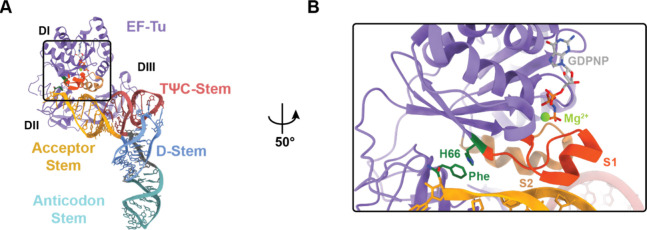
Structure of the ternary complex. (A) Crystal structure of the ternary complex (PDB: 1OB2), highlighting the different structural domains of tRNA (variegated) and EF-Tu (purple). (B) Zoomed in image of the boxed area in (A), showing the EF-Tu amino acid binding pocket and highlighting specific functional elements for ternary complex stability, such as the π–π stacking interaction between phenylalanine (Phe; green) aminoacylated to the 3′ end of tRNA^Phe^ (yellow), histidine 66 (H66; green), and the switch 1 (S1; orange) and switch 2 (S2; tan) helices that coordinate to the GTP in the EF-Tu nucleotide binding pocket. The coordinated magnesium (Mg^2+^) is shown in light green, and the nonhydrolyzable GDPNP analogue is shown in gray.

During translation, aa-tRNAs are delivered to the ribosome in the ternary complex with thermally unstable elongation factor (EF-Tu) (eEF1A in eukaryotes) and GTP. The three domains (DI–III) of EF-Tu directly engage the amino acid backbone and side chain as well as the TψC and acceptor stems of tRNA to form a high-affinity (ca. 10–100 nM) complex (Figure 1A).^17−19^ EF-Tu exhibits distinct affinities for each aa-tRNA species (−8 to −11 kcal/mol).^20−22^ The prevailing hypothesis is that this variance ensures uniform decoding speeds by balancing the spring-like forces that accumulate in aa-tRNA during initial selection and proofreading steps of tRNA selection on the ribosome that ultimately lead to aa-tRNA dissociation from EF-Tu, allowing the aminoacylated 3′-CCA terminus of the tRNA to enter the ribosome’s peptidyltransferase center.^23−25^ During proofreading selection, which occurs after GTP hydrolysis at the end of initial selection, rate-limiting conformational changes in EF-Tu that are responsible for triggering its dissociation from aa-tRNA and the ribosome contribute to substrate discrimination by allowing additional time for near- and noncognate aa-tRNAs to dissociate.^23,26−31^ Together, initial selection and proofreading selection ensure an error rate of approximately one in 1000–10 000 mRNA codons for natural α-amino acids.^32−34^

The active (GTP-bound) form of EF-Tu harbors a precisely formed binding pocket for the α-amino acid backbone as well as a relatively spacious cavity for the side chains of the 20 naturally occurring amino acids. Upon aa-tRNA engagement, all three EF-Tu domains (DI–III) collapse around the CCA-3′ end of the tRNA acceptor stem to position the constituent components of the amino acid side chain, which concomitantly restructures the switch 1 (S1) and switch 2 (S2) helices to engage the γ-phosphate of GTP via Mg^2+^ coordination to yield the thermodynamically stable ternary complex (Figure 1B).^35,36^ This stability strictly depends on tRNA aminoacylation and the presence of a properly positioned γ-phosphate moiety.^19,37^ In line with this exquisite sensitivity, in vitro transcribed tRNAs misacylated with native amino acids can exhibit binding affinities for EF-Tu(GTP) that are reduced by up to ∼5000-fold,^21^ which affect both the speed and fidelity of tRNA selection on the ribosome.^38−40^ However, the precise contributions of the amino acid backbone to the EF-Tu(GTP) affinity have yet to be fully explored.

In bacteria, ternary complex formation is catalyzed by the conserved EF-Tu-specific guanosine nucleotide exchange factor (GEF), EF-Ts. Under nutrient-rich conditions where GTP concentration far exceeds that of GDP, EF-Ts ensures rapid and abundant ternary complex formation so that it is not rate-limiting to protein synthesis.^19,37^ Under nutrient-poor conditions where GDP concentrations are elevated, EF-Ts instead facilitates ternary complex disassembly, reducing protein synthesis and other energy intensive cellular processes.^19,37^ The impact of EF-Ts on both ternary complex formation and dissociation indicates that nucleotide exchange and S1 restructuring are dynamic processes that can be influenced by EF-Ts in both the absence and presence of aa-tRNA.

Here we use ensemble and single-molecule fluorescence resonance energy transfer (FRET)-based kinetic assays, together with kinetic simulations, to investigate the effects of both non-natural α-amino acids (specifically, those with a non-natural side chain and a natural α-backbone) and non-natural backbones (specifically, those with a natural side chain and a d-α- or a non-natural β^2^- or β^3^-amino acid backbone) on the kinetics and thermodynamics of ternary complex formation and tRNA elongation on the ribosome.^19,37^ Consistent with its widespread use by diverse research laboratories, our investigations show that the metrics of both ternary complex formation and tRNA selection are virtually identical for tRNAs acylated with l-α-Phe or the non-natural α-amino acid *para*-azido-phenylalanine (*p*-Az-Phe). By contrast, the kinetics of both ternary complex formation and tRNA selection were altered for backbone-modified monomers. The presence of either d-α-Phe, (*R*)-, or (*S*)-β^2^-Phe in place of l-α-Phe disrupts ternary complex formation to levels below our in vitro detection limits, whereas (*R*)- and (*S*)-β^3^-Phe reduce ternary complex stability by approximately an order of magnitude. These deficiencies are exacerbated by EF-Ts, and by mutations in both EF-Tu and tRNA previously reported to stabilize ternary complex using in vitro transcribed tRNAs.^41,42^ In line with these observations, ribosomes fail to recognize d-α-Phe-, (*R*)-, and (*S*)-β^2^-Phe-charged tRNAs as substrates, while the utilization of (*R*)- and (*S*)-β^3^-Phe stereoisomers was significantly impaired relative to native l-α-Phe. The reduced EF-Tu affinities of tRNAs acylated with either (*R*)- or (*S*)-β^3^-Phe also precipitated defects in the mRNA decoding mechanism, where the proofreading stage of the tRNA selection process immediately after GTP hydrolysis was ostensibly bypassed. Following incorporation into the ribosome, tRNAs charged with d-α-Phe, (*R*)-, and (*S*)-β^3^-Phe stereoisomers were both competent for translocation. However, as predicted using recent metadynamics simulations,^16^ (*R*)-β^3^-Phe appeared to exhibit order of magnitude defects in the rate of peptide bond formation, which dramatically reduced the rate of substrate translocation from hybrid-state tRNA positions. We conclude from these findings that the efficiency of ternary complex formation and its thermodynamic stability are key determinants of nnAA incorporation into proteins and that engineering opportunities exist to enable their more efficient utilization.

## Results

### Quantifying Ternary Complex Formation

**Figure 2:**
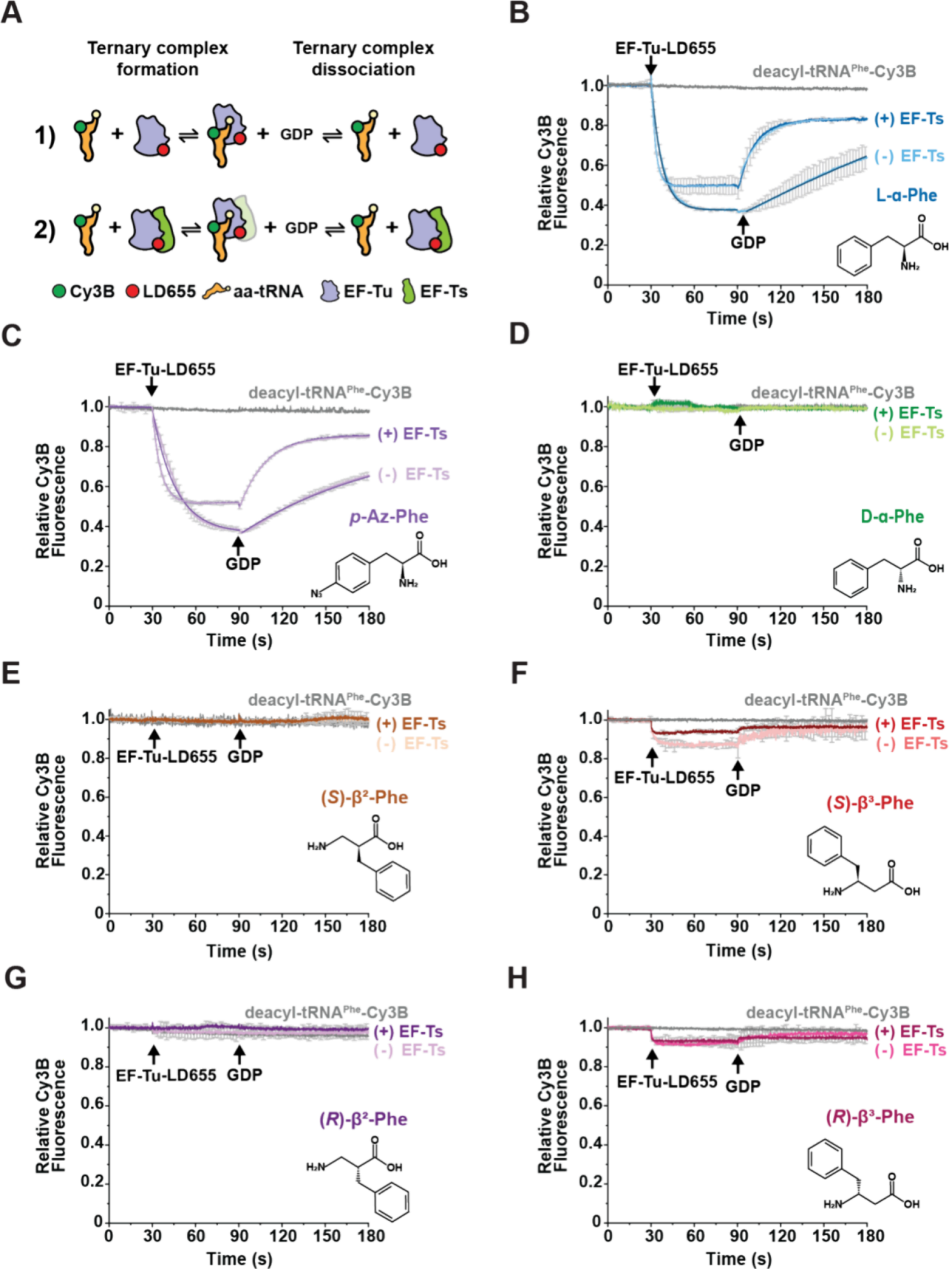
Ensemble FRET-based assay to measure ternary complex formation and dissociation. (A) Cartoon schematics of the FRET-based ensemble ternary complex formation assay, where LD655-labeled EF-Tu (blue) (1) without or (2) with EF-Ts (green) (400 nM) is injected into a cuvette containing 5 nM aa-tRNA^Phe^-Cy3B (orange) in the presence of 10 μM GTP. Formation of ternary complex results in rapid quenching of Cy3B fluorescence via FRET that can be recovered upon dissociation after injection of 100 μM GDP. In (2), due to the experimental conditions used (labeling strategy and 100 ms time resolution), the transient formation of the previously reported quaternary complex (EF-Tu/Ts·GTP·aa-tRNA) could not be observed. (B–H) Ensemble ternary complex assays tracking Cy3B relative fluorescence changes over time upon mixing LD655-labeled EF-Tu (+ EF-Ts), and subsequent addition of GDP, with tRNA^Phe^-Cy3B aminoacylated with (B) l-α-Phe, (C) *p*-Az-Phe, (D) d-α-Phe, (E) (*S*)-β^2^-Phe, (F) (*S*)-β^3^-Phe, (G) (*R*)-β^2^-Phe, and (H) (*R*)-β^3^-Phe. Monomer structures are shown to the right of each plot. Plots represent mean ± SD from two experimental replicates.

To directly investigate how various types of non-natural amino acids impact the kinetic features of ternary complex formation, we employed an ensemble approach in which the rate and extent of ternary complex formation was tracked via FRET.^19,37^ In this assay, EF-Tu carrying an LD655 fluorophore at its C-terminus quenches the fluorescence of a Cy3B fluorophore attached to the 3-amino-3-carboxypropyl (acp^3^) modification at position U47 of native tRNA^Phe^ when the ternary complex forms (Figure 2A). Reactions were initiated upon the stopped-flow addition of 400 nM EF-Tu-LD655 to a solution of 5 nM l-α-Phe-tRNA^Phe^-Cy3B in a 1.2 mL cuvette with stirring at 25 °C (see Experimental Procedures section). Under these conditions, the observed pseudo-first-order apparent rate constant (*k*_obs_) for Cy3B fluorescence quenching was 0.12 ± 0.002 s^–1^, reaching ∼65% quenching at equilibrium (Figure 2B and Table 1). The observed fluorescence quenching amplitude agrees with the close proximity of Cy3B and LD655 fluorophores within the ternary complex (Figure 1).^19^ Consistent with the GTP requirement for ternary complex formation, the rapid stopped-flow addition of 100 μM GDP to the reaction restored Cy3B fluorescence to ∼80% of the initial intensity with an apparent rate constant (*k*_off_) of 0.005 ± 0.001 s^–1^ (Figure 2B and Table 1). Identical experiments performed using deacyl-tRNA^Phe^-Cy3B in place of l-α-Phe-tRNA^Phe^-Cy3B resulted in no Cy3B quenching (Figure 2B), which is congruous with the selectivity of EF-Tu for aminoacylated tRNA.

**Table 1:** Apparent Reaction Rates Estimated by Single-Exponential Fitting of the Ensemble Ternary Complex Formation Experiments Shown in Figure 2^a^

observed rates	l-α-Phe		*p*-Az-Phe	
EF-Ts	–	+	–	+
*k*_obs_ (s^–1^)	0.12 ± 0.002	0.31 ± 0.04	0.06 ± 0.01	0.25 ± 0.02
*k*_off_ (s^–1^)	0.005 ± 0.001	0.06 ± 0.007	0.01 ± 0.0004	0.06 ± 0.01

a Uncertainty estimates represent SD from two experimental replicates. Single-exponential fits exhibited *R*^2^ values >0.95. The l-α-Phe rates compare well with previously reported results.^37^

When analogous experiments were performed with the nucleotide exchange factor EF-Ts present in a 1:1 ratio relative to EF-Tu, *k*_obs_ increased to 0.31 ± 0.04 s^–1^, and the extent of Cy3B fluorescence quenching at equilibrium was reduced to ∼50%. The addition of 100 μM GDP to the same reaction mixture restored the Cy3B fluorescence amplitude with a *k*_off_ that was approximately 10-fold faster than with EF-Tu alone (0.06 ± 0.007 s^–1^). As for EF-Tu alone, the fluorescence intensity prior to ternary complex formation was restored, although not to the full extent due to residual ternary complex formation under equilibrium conditions (Figure 2A,B). These observations are consistent with previously reported affinities of EF-Tu(GTP) for aa-tRNA (ca. 10–100 nM) and prior conclusions that EF-Ts can engage with EF-Tu in ternary complex to catalyze nucleotide exchange, thereby accelerating ternary complex dissociation rates after GDP addition by ∼20-fold.^19,37^

### Ternary Complex Stability Is Disrupted by d-α- and β-Phe Monomers

With this foundation, we next asked how ternary complex stability and rates of formation were affected when tRNA^Phe^-Cy3B was aminoacylated with nnAAs such as *p*-Az-Phe, d-α-Phe, (*R*)- and (*S*)-β^2^-Phe, and (*R*)- and (*S*)-β^3^-Phe. These nnAA monomers were used to aminoacylate native tRNA^Phe^-Cy3B using the previously reported flexizyme system.^43−45^ Each aa-tRNA^Phe^-Cy3B species was purified by fast protein liquid chromatography (FPLC), flash frozen in aliquots, stored at −80 °C, and verified as being at least 90% aminoacylated prior to use (Experimental Procedures and Figure S1). We confirmed that flexizyme-charged l-α-Phe-tRNA^Phe^-Cy3B exhibited nearly identical formation/dissociation kinetics and fluorescence quenching/recovery amplitudes as the enzymatically charged species (Figure S2), validating this experimental tRNA aminoacylation strategy for kinetic assays.^6,15,44,46^

Using experimental conditions identical to those described above, we next examined the kinetics of ternary complex assembly using tRNA^Phe^-Cy3B that was acylated with *p*-Az-Phe, a nnAA monomer successfully incorporated into proteins by multiple laboratories.^10,13,47,48^ The stopped-flow addition of EF-Tu-LD655 to *p-*Az-Phe-tRNA^Phe^-Cy3B resulted in Cy3B fluorescence quenching characterized by a *k*_obs_ of 0.06 ± 0.01 s^–1^; the extent of fluorescence quenching reached ∼65% at equilibrium (Figure 2C and Table 1). As observed for the complex of l-α-Phe-tRNA^Phe^-Cy3B, the rapid addition of GDP slowly restored Cy3B fluorescence; in this case, the measured *k*_off_ was 0.01 ± 0.0004 s^–1^ (Figure 2C and Table 1). In the presence of EF-Ts, ternary complex formation again proceeded more rapidly (*k*_obs_ = 0.25 ± 0.02 s^–1^), and the fluorescence quenching reached an amplitude of ∼50% at equilibrium (Figure 2C and Table 1). Ternary complex dissociation upon the addition of GDP proceeded with a *k*_obs_ of 0.06 ± 0.02 s^–1^, restoring Cy3B fluorescence to ∼80% of the initial intensity. Overall, the kinetic parameters measured for assembly and disassembly of ternary complexes containing *p-*Az-Phe-tRNA^Phe^-Cy3B were similar to those measured for complexes containing l-α-Phe-tRNA^Phe^-Cy3B. Thus, the presence of the *p*-Azido side chain on Phe has, as expected, only a modest impact on ternary complex formation and stability. These results are consistent with the functionalized phenyl side chain being readily accommodated by EF-Tu.

We next investigated the kinetics of ternary complex assembly and disassembly when tRNA^Phe^-Cy3B was aminoacylated with d-α-Phe and the (*R*)- or (*S*)- enantiomers of β^2^- or β^3^-Phe. Many d-α- and β^2^- and β^3^-amino acids have been introduced into short peptides using small-scale in vitro translation reactions,^49,50^ and a few β^3^-amino acids have been introduced into proteins in cell lysates.^51,52^ Moreover, one β^3^-Phe derivative,^6^ three β^3^-aryl derivatives,^8^ and one β^2^-hydroxy acid^7^ have been introduced into proteins in cells. Yet in none of these cases was the level of incorporation especially high, perhaps because of impaired delivery to the ribosome by EF-Tu.^53,54^

In support of this hypothesis, we detected no evidence for ternary complex formation in reactions containing d-α-Phe-, (*S*)-, or (*R*)-β^2^-Phe-tRNA^Phe^-Cy3B (Figure 2D,E,G), while reactions containing (*S*)- or (*R*)-β^3^-Phe-tRNA^Phe^-Cy3B exhibited detectable, yet greatly reduced, levels of Cy3B fluorescence quenching (∼9–15% vs ∼60–65% with l-α-Phe) (Figure 2F,H). Remarkably, (*S*)-β^3^-Phe-tRNA^Phe^-Cy3B showed a higher degree of Cy3B quenching than (*R*)-β^3^-Phe-tRNA^Phe^-Cy3B (14% vs 9%), mirroring their degrees of structural similarity to l-α-Phe. Quenching was reversed upon the addition of 100 μM GDP, as expected for ternary complex disassembly (Figure 2A). The addition of EF-Ts to the ternary complex assembly reactions containing (*R*)- or (*S*)-β^3^-Phe attenuated Cy3B fluorescence quenching, consistent with decreased ternary complex stability.^19,37^ These observations suggest that the opposite chirality of d-α-Phe and the extended backbones of β^2^- and β^3^-Phe perturb the interface with EF-Tu to reduce its capacity to stably engage aa-tRNA^Phe^. However, due to the impaired signal amplitudes of these reactions, reliable *k*_obs_ and *k*_off_ rates could not be estimated.

### β^2^-Phe and β^3^-Phe Monomers Negatively Impact the Kinetic Features of Ternary Complex Formation

**Figure 3:**
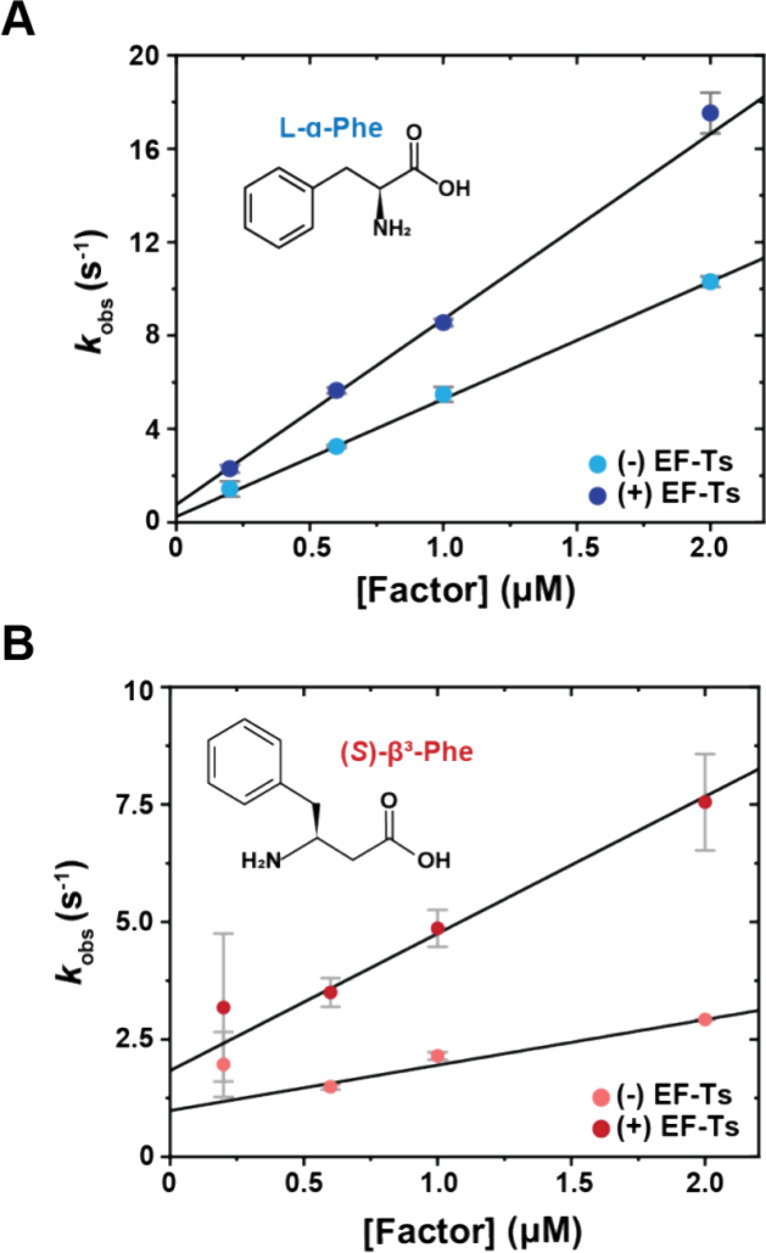
Determination of microscopic rate constants of ternary complex formation from rapid, pre-steady-state measurements. (A) Plot showing the apparent rate (*k*_obs_) of ternary complex formation for l-α-Phe-tRNA^Phe^-Cy3B as a function of EF-Tu (sky blue) and EF-Tu/Ts (navy blue) concentrations, performed in the presence of 100 μM GTP. (B) Analogous plot of *k*_obs_ for (*S*)-β^3^-Phe-tRNA^Phe^-Cy3B in the absence (pink) and presence (red) of equimolar EF-Ts. Each data point represents mean ± SD from 3–7 experimental replicates.

We next employed a microvolume stopped-flow system (μSFM, Biologic; see Experimental Procedures section) to perform a thorough kinetic study of ternary complex formation for tRNAs carrying (*R*)- or (*S*)-β^3^-Phe, which showed the highest fluorescence quenching of the four acylated tRNAs studied above. Using this approach, we measured bimolecular association rate constants for the binding of EF-Tu to tRNA^Phe^-Cy3B acylated with either l-α-Phe- or (*S*)-β^3^-Phe-charged tRNA^Phe^ by tracking how *k*_obs_ changed as a function of the concentration of EF-Tu or EF-Tu/Ts. Upon rapid mixing of 5 nM l-α-Phe-charged tRNA^Phe^-Cy3B with a large excess of EF-Tu (0.2–2 μM) and GTP (1 mM), we observed a linear increase in *k*_obs_ as a function of both [EF-Tu] and [EF-Tu/Ts], consistent with pseudo-first-order binding kinetics (Figure 3A,B). In line with previous literature,^19,37^ in the absence of EF-Ts, the bimolecular rate constant (*k*_on_) was 5.0 ± 0.1 μM^–1^ s^–1^, and the dissociation rate constant (*k*_off_) was 0.2 ± 0.1 s^–1^ (Table 2). With equimolar EF-Tu/Ts, *k*_on_ and *k*_off_ increased by 1.6- and 4-fold, respectively.^19,37^ Consistent with our initial findings, when l-α-Phe was substituted with (*S*)-β^3^-Phe, we observed both a lower *k*_on_ (1.0 ± 0.1 μM^–1^ s^–1^) and a higher *k*_off_ (1 ± 0.1 s^–1^) (Table 2). In the presence of EF-Ts, *k*_on_ and *k*_off_ increased by 3- and 1.8-fold, respectively (Table 2). For (*R*)-β^3^-Phe, the fluorescence intensity changes were still too small for reliable estimations of its kinetic parameters (Figure S3). Additionally, we were unable to rescue the observed defects using tRNA (C49A, G65U) and EF-Tu (N273A) mutations previously reported to stabilize the ternary complex formation (Experimental Procedures and Figure S3). We hypothesize that disparities between our results and previous studies in this regard stem from differences in experimental design that is most likely attributed to the use of in vitro transcribed tRNA instead of native species and EF-Tu from the hyperthermophile *Thermus thermophilus* instead of *E. coli* as well as the methods employed (RNase protection and electrophoretic mobility shift assays vs FRET-based assays).^41,42^

**Table 2:** Kinetic and Thermodynamic Parameters for aa-tRNA^Phe^ Binding to EF-Tu-LD655 in the Presence and Absence of EF-Ts from the Experiments in Figure 3^a^

rate constant	l-α-Phe		(*S*)-β^3^-Phe	
EF-Ts	–	+	–	+
*k*_on_ (μM^–1^ s^–1^)	5.0 ± 0.1	8.0 ± 0.3	1.0 ± 0.1	3.0 ± 0.3
*k*_off_ (s^–1^)	0.2 ± 0.1	0.8 ± 0.2	1.0 ± 0.1	1.8 ± 0.2
*K*_D_ (est.)	40 nM	100 nM	1 μM	0.6 μM
Δ*G°* (kcal/mol)	–11.5	–9.6	–4.1	–4.4

a *K*_D_ values were estimated from the ratio *K*_D_ = *k*_off_ /*k*_on_, determined from titration experiments in Figure 3. Free energies were calculated using the relationship Δ*G°* = *–RT* ln(1/*K*_D_)^21^.

From these findings, we estimated equilibrium dissociation constants (*K*_D_) for the ternary complexes of EF-Tu with l-α-Phe-tRNA^Phe^-Cy3B of approximately 40 and 100 nM in the absence and presence of EF-Ts, respectively (Table 2). Similar analyses estimated ternary complex *K*_D_ values of ∼1000 and ∼600 nM for the analogous complexes containing (*S*)-β^3^-Phe in the absence and presence of EF-Ts, respectively (Table 2). From these *K*_D_ estimates, we calculated Δ*G°* values of −9 to −11 kcal/mol for complexes containing l-α-Phe, consistent with previous literature,^21,42,55^ while the Δ*G°* values for complexes containing (*S*)-β^3^-Phe were only −4.1 to −4.4 kcal/mol. Hence, the stability of the (*S*)-β^3^-Phe-containing ternary complex would be outside of the thermodynamic range for ternary complexes that support efficient translation, as both tightly and loosely bound aa-tRNAs to EF-Tu impair translation by slowing peptide bond formation^56^ or aa-tRNA delivery to the ribosome, respectively^55^ (Table 2).

These data support the hypothesis that both β^2^- and β^3^-Phe monomers interfere with EF-Tu engagement by reducing both the efficiency with which EF-Tu productively engages acyl-tRNA as well as the overall stability of the ternary complex. Despite an approximate 25-fold reduction in affinity for (*S*)-β^3^-Phe-tRNA^Phe^, kinetic simulations based on a simplified framework (Figure S4A), suggest that (*S*)-β^3^-Phe-containing ternary complexes can be populated significantly in a cellular context, where EF-Tu and EF-Ts are present at ∼μM concentrations (Figure S4B,C).^57^ We speculate that increasing cellular EF-Tu concentrations may only partially alleviate incorporation deficiencies due to complex instability.

### β-Phe-Charged tRNAs Are Poorly Accommodated on the Ribosome and Ostensibly Bypass Proofreading Steps

**Figure 4:**
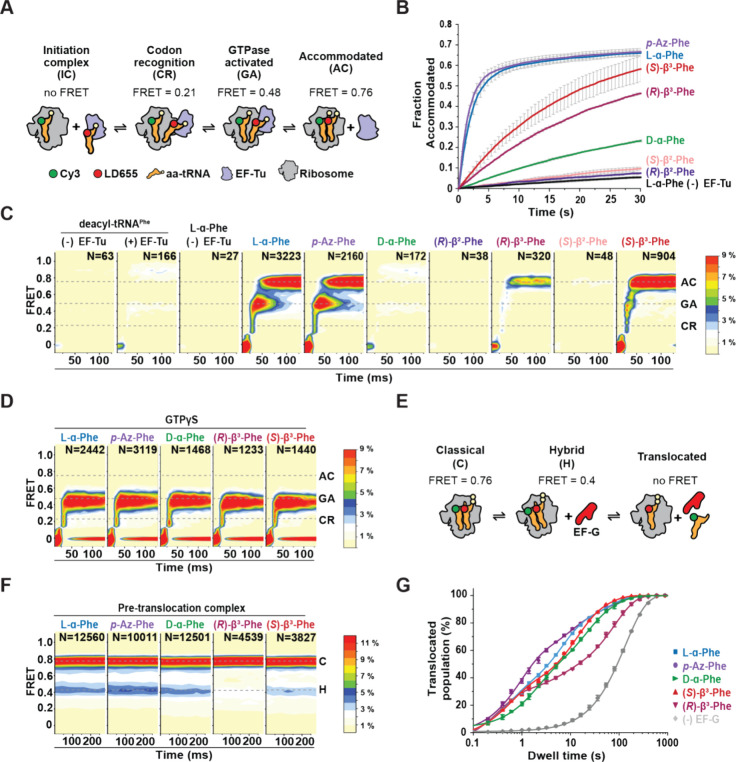
nnAA-tRNA selection on the bacterial ribosome studied by smFRET. (A) Schematic describing the smFRET tRNA selection experimental design, where LD655-labeled aa-tRNA^Phe^ is delivered to surface-tethered ribosomes (blue) bearing Cy3-fMet-tRNA^fMet^ in the P site. tRNA selection intermediates and the fully accommodated tRNA are distinguished based on their distinct FRET values. (B) Fraction of ribosomes bearing a fully accommodated aa-tRNA as a function of time (Experimental Procedures). Data were collected at 100 ms time resolution; experiments were performed in triplicate. (C, D) 2D histograms of smFRET traces containing productive accommodation events with GTP (C) or stalled events with GTPγS (D) (see Experimental Procedures section) (*N*, number selected), postsynchronized to the appearance of FRET above baseline, with the specified amino acids. For clarity, contour plots (except for l-α-Phe and *p*-Az-Phe) were normalized (scale at right) to the (*S*)-β^3^-Phe trace number. Dashed lines indicate FRET efficiency values of tRNA selection intermediates used in kinetic modeling. Data were collected at 10 ms time resolution. (E) Schematic describing the smFRET translocation experimental design. (F) 2D histograms of smFRET traces of accommodated aa-tRNA after buffer exchange and ∼5 min equilibration time, showing both classical (accommodated) and hybrid (P/E, A/P) tRNA conformations. Data were collected at 40 ms time resolution. (G) Dwell time distribution of one full elongation cycle. Lines represent fits to one (l-α-Phe without EF-G) or three (l-α-Phe, *p*-Az-Phe, (*R*)- and (*S*)-β^3^-Phe) exponential distributions (Experimental Procedures). Three different dwell time regimes are observed: the hybrid state (∼0.5 s), the classical state (∼8 s), and the “slow” state (∼60 s). Data were collected at 200 ms time resolution. For all tRNA selection experiments, LD655-labeled aa-tRNA^Phe^ and EF-Tu/Ts concentrations were 12.5 and 125 nM, respectively. For all elongation experiments, LD655-labeled aa-tRNA^Phe^ and EF-Tu/Ts concentrations were 25 and 250 nM, respectively.

To examine how ternary complexes formed with nnAAs are incorporated by the ribosome, we employed single-molecule FRET (smFRET) imaging methods to directly monitor both the frequency of productive ternary complex binding to the ribosome as well as the process by which an aa-tRNA is released from EF-Tu and incorporated into the ribosomal aminoacyl (A) site.^23,25,30^ This established approach tracks FRET between a donor fluorophore linked to a peptidyl-tRNA bound within the ribosomal P site and an acceptor fluorophore linked to the incoming aa-tRNA (Figure 4A).

To perform these studies, bacterial ribosome complexes were programmed with a synthetic 5′-biotinylated mRNA that positions fMet-tRNA^fMet^-Cy3 in the P site and a UUC codon in the A site. Initiation complexes were then tethered via a biotin–streptavidin bridge proximal to an optically transparent, poly(ethylene glycol) (PEG)-passivated surface. Single-molecule tRNA selection experiments were subsequently initiated by the stopped-flow injection of ternary complexes formed with flexizyme-charged tRNA^Phe^-LD655, where EF-Tu/Ts (125 nM) is in 10-fold excess over aa-tRNA (12.5 nM) (Experimental Procedures).

We first assessed the apparent rates and extents of ternary complex utilization by the ribosome by collecting pre-steady-state movies at low time resolution (100 ms/video frame), where photobleaching is minimized. Consistent with our ensemble ternary complex formation studies (Figures 2 and 3), *p*-Az-Phe-tRNA^Phe^-LD655 was utilized as efficiently as l-α-Phe-tRNA^Phe^-LD655 (Figure 4B and Table 3). Both (*R*)- and (*S*)-β^2^-Phe-tRNA^Phe^-LD655 were not utilized by the ribosome, while d-α-Phe- and both (*R*)- and (*S*)-β^3^-Phe-tRNA^Phe^-LD655 were utilized >10-fold less compared to l-α-Phe-tRNA^Phe^-LD655. We attribute these observations to defects in the formation of ternary complexes (Figure 4B and Table 3).

**Table 3:** Apparent aa-tRNA Accommodation Rates on the Ribosome Calculated from the Experiments in Figure 4^a^

amino acid	*k*_1 obs_ (s^–1^)	*k*_2 obs_ (s^–1^)
l-α-Phe (−) Tu-Ts	0.025 ± 0.002	N/A
l-α-Phe	0.46 ± 0.04	0.033 ± 0.001
*p*-Az-Phe	0.59 ± 0.03	0.036 ± 0.002
d-α-Phe	0.03 ± 0.001	N/A
(*R*)-β^2^-Phe	0.024 ± 0.003	N/A
(*R*)-β^3^-Phe	0.041 ± 0.002	N/A
(*S*)-β^2^-Phe	0.024 ± 0.001	N/A
(*S*)-β^3^-Phe	0.056 ± 0.006	N/A

a Uncertainty estimates represent SD from three experimental replicates.

To gain more specific insights into the tRNA selection mechanism after ternary complex binding to the ribosome, we performed analogous pre-steady-state experiments at 10-fold higher time resolution (10 ms/video frame). As previous smFRET studies of bacterial tRNA selection have shown,^23,30^ experiments of this kind allow for the direct assessment of codon recognition (CR), GTPase activation (GA), and accommodation (AC), as each reaction end point exhibits a distinct FRET efficiency value (∼0.21, ∼ 0.48, and ∼0.76, respectively; see Experimental Procedures section). Apparent initial binding rates, as measured by the time delay between stopped-flow ternary complex delivery and the first detected FRET event, directly report on ternary complex concentration. For l-α-Phe and *p*-Az-Phe, we found that the arrival rate constant was 1.3 ± 0.2 and 1.9 ± 0.2 s^–1^, respectively, consistent with the bimolecular rate constant of ∼150 μm^–1^ s^–1^ shown previously.^23^ For d-α-Phe and the four β^3^-Phe monomers, the apparent rates were approximately 2-fold lower (0.6–0.9 s^–1^) (Figure S5A). These rate constants are indistinguishable from the arrival rate constant of deacyl-tRNA^Phe^ (both in the absence and presence of EF-Tu/Ts) (Figure S5A), confirming each monomer’s reduced ternary complex stability and in line with our ensemble FRET-based measurements. By computationally isolating FRET trajectories reflecting productive ternary complex binding events to individual ribosomes (Experimental Procedures)^23^ (i.e., those that stably accommodate) (Figure 4C), we found that l-α-Phe and *p-*A*z*-Phe exhibited similar progression probabilities through tRNA selection, including the rate-limiting progression from the GTPase-activated state (∼0.48, GA) to the fully accommodated state (∼0.76, AC) during proofreading selection. Consistent with our ensemble and low-time-resolution smFRET assays, successful tRNA selection events were ostensibly not observed for (*R*)- or (*S*)-β^2^-Phe, whereas we observed ∼5–10-fold fewer productive tRNA selection events for d-α-Phe and (*R*)- and (*S*)-β^3^-Phe ternary complexes (Figure 4C). Strikingly, these rare events of (*R*)- and (*S*)-β^3^-Phe tRNA accommodation exhibited much more rapid passage of the intermediate states of tRNA selection compared to l-α-Phe. Quantitative kinetic analysis revealed that the forward transit time from GA to AC for l-α-Phe and *p*-Az-Phe was 33 ± 0.5 and 35 ± 0.6 ms, respectively, comparable to previous studies^23,30^ (Figure S5B). In contrast, the forward transit time for (*R*)- and (*S*)-β^3^-Phe was at least 3-fold shorter (<10 ms), falling below the time resolution of our measurements (Figure S5B). Notably, the (*R*)- and (*S*)-β^2^-Phe forward transit time was 3-fold longer, and that for d-α-Phe was 10-fold longer. The backward transit time from GA to CR was similar for all monomers studied (17–33 ms), with the exception of d-α-Phe (99 ± 16 ms) (Figure S5B). Consequently, the probability of successfully accommodating from the GA state for l-α-Phe, *p*-Az-Phe, and (*S*)-β^3^-Phe was ∼0.45, while it was ∼0.29 for (*R*)-β^3^-Phe. Due to their longer forward transit times, the accommodation probability for d-α-Phe, (*R*)-, and (*S*)-β^2^-Phe was ∼0.05 (Figure S5C), resulting in a significant reduction in the number of successful accommodation events. To test whether the tRNA selection process for (*R*)- and (*S*)-β^3^-Phe bypassed GTP hydrolysis, we performed identical smFRET experiments with the nonhydrolyzable GTPγS analogue to stall tRNA selection at the GTP hydrolysis step at the end of initial selection.^37^ In the presence of GTPγS, both (*R*)- and (*S*)-β^3^-Phe were efficiently stalled in the GA state, as were l-α-Phe, d-α-Phe, and *p*-Az-Phe (Figure 4D). These findings reveal that GTP hydrolysis is indeed required for (*R*)- and (*S*)-β^3^ for GA-state passage and the proofreading process. The observation that proofreading is significantly more rapid for (*R*)- and (*S*)-β^3^-Phe-tRNA^Phe^ (see above) supports the notion that conformational changes in EF-Tu during proofreading related to 3′-CCA release, e.g., disordering of the S1 helix, are rate-limiting to the tRNA selection process^23,30^ and that this impact can be specifically attributed to the β^3^-Phe monomers. Reduced thermodynamic stability leads to more rapid EF-Tu dissociation from aa-tRNA and likely the ribosome, decreasing the GA lifetime (Figure S5B).

### β^3^-Phe-Charged tRNAs Impair Elongation Cycle Steps after tRNA Selection

Since we were able to observe some β^3^-Phe accommodation on the ribosome, we next wanted to assess if (*R*)- and (*S*)-β^3^-Phe-tRNA^Phe^ impact later steps of the elongation cycle, including peptide bond formation and EF-G-catalyzed translocation (Figure 4E). To examine these steps, first we quantified the equilibrium distribution between classical (∼0.76, C) and hybrid (∼0.4, H) state pretranslocation complex conformations that are adopted spontaneously after tRNA selection is complete^58−60^ (Figure 4F). l-α-Phe, *p*-Az-Phe, and d-α-Phe showed a 64:36 ±2 ratio between classical and hybrid pretranslocation complexes. Notably, (*S*)-β^3^-Phe did not significantly affect the classical–hybrid ratio (67:33 ± 3), whereas (*R*)-β^3^-Phe preferentially adopted classical conformations (74:26 ± 2; *p* < 0.05; Experimental Procedures). These results indicate that, while (*S*)-β^3^-Phe behaves similarly to its α-amino acid counterparts, (*R*)-β^3^-Phe also perturbs spontaneous transitions to translocation-ready hybrid states.^24^ Hence, the incorporation of (*R*)-β^3^-Phe into proteins is predicted to increase the frequency of elongation pauses.

We next performed analogous smFRET experiments under cell-like conditions, where productive tRNA selection events are rapidly followed by translocation. To initiate the complete elongation cycle, we stopped-flow injected ternary complexes (250 nM EF-Tu/Ts, 25 nM aa-tRNA^Phe^-LD655) together with EF-G (8 μM) in the presence of 1.25 mM GTP. In this experiment, the appearance of FRET reports on aa-tRNA incorporating into the ribosome. The loss of FRET reports on the dissociation of Cy3-labeled initiator tRNA^fMet^ from the E site during, or after, the process of translocation^61^ (Figure 4E,G). Correspondingly, the FRET lifetime in these experiments reports on the total duration of the elongation cycle. We measured complete elongation cycle times using ternary complexes formed with l-α-Phe, *p*-Az-Phe, d-α-Phe, and (*R*)- and (*S*)-β^3^-Phe while including a control study lacking EF-G to estimate the contribution of fluorophore photobleaching (Figure 4F). Consistent with each complex undergoing EF-G-catalyzed translocation, the average FRET lifetimes were substantially shorter (ca. 2.5–8-fold) than photobleaching (165.8 ± 2.8 s). In line with prior investigations of translocation,^24,61−65^ the FRET lifetime distributions for each amino acid type displayed multimodal behaviors, characterized by a very fast (∼0.3 s), fast (∼5–10 s), and slow (∼30–40 s) kinetic subpopulations (Table 4). We attribute these subpopulations to pretranslocation complexes that undergo rapid translocation from hybrid-state conformations, classical pretranslocation complexes that must wait for spontaneous transitions to hybrid-state conformations, and pretranslocation complexes that either exhibit more substantial delays in translocation (“slow”) due to functional defects or retain deacyl-tRNA in the E site after translocation is complete, respectively. All monomers tested exhibit fast translocating subpopulations, consistent with rapid peptide bond formation followed by quick translocation from hybrid-state conformations. Subpopulations were also present for all five monomers that exhibited long-lived FRET lifetimes, consistent with relatively stable, classical ribosome conformations requiring additional time to first transition to hybrid states before being able to productively engage EF-G and then rapidly translocate^24,61,63^ (Table 4). We note in this context that d-α-Phe showed a 2-fold increased FRET lifetime for the very fast kinetic subpopulation compared to all other monomers. Moreover, (*R*)-β^3^-Phe, the monomer most dissimilar to l-α-Phe, exhibited a relatively large subpopulation of pretranslocation complexes that exhibit slow translocation, which may reflect specific deficiencies in peptide bond formation, hybrid-state formation, or deacyl-tRNA release from the E site.

**Table 4:** Dwell Times for Hybrid, Classical, and “Slow” States from the Fittings to Three Exponential Distributions (Experimental Procedures)^a^

	hybrid state		classical state		“slow” state	
amino acid	dwell time (s)	population (%)	dwell time (s)	population (%)	dwell time (s)	population (%)
l-α-Phe	0.44 ± 0.11	30.1 ± 3.6	6.12 ± 0.68	48.2 ± 3.1	59.17 ± 3.12	21.7 ± 1.7
*p*-Az-Phe	0.87 ± 0.08	53.3 ± 2.2	8.45 ± 1.13	26.2 ± 1.6	67.46 ± 3.35	20.5 ± 1.2
d-α-Phe	1.76 ± 0.10	37.1 ± 1.3	16.91 ± 2.05	40.4 ± 3.6	70.05 ± 10.38	22.5 ± 4.3
(*S*)-β^3^-Phe	0.53 ± 0.07	33.6 ± 2.9	11.81 ± 0.83	46.7 ± 2.2	47.56 ± 3.76	19.7 ± 2.5
(*R*)-β^3^-Phe	0.33 ± 0.03	33.9 ± 0.9	4.67 ± 0.80	12.1 ± 1.2	80.79 ± 3.31	54.0 ± 1.1

a Photobleaching lifetime is 165.8 ± 0.03 s.

## Discussion

Efficient tRNA selection by the ribosome is paramount to the transfer of genetic information from mRNA to protein.^66^ Harnessing this template-driven platform through genetic code expansion to synthesize previously unknown and useful biopolymers promises myriad tools to advance research and medicine.^11^ Achieving this goal requires continual technology development to overcome bottlenecks that limit the efficiency of non-α-amino acid incorporation. These bottlenecks in principle include, but are not limited to, differences in monomer uptake and cellular stability, aminoacyl-tRNA synthetase activity and fidelity, ternary complex formation of the aa-tRNA with EF-Tu(GTP), constraints present within the ribosome PTC itself, and other fidelity mechanisms evolved to reduce erroneous amino acid incorporation by the ribosome.^13,67−70^ Here we employ both ensemble and single-molecule kinetic assays to show that the formation of ternary complexes and utilization by the ribosome represent significant bottlenecks for the use of d-α-Phe and β-Phe monomers.

tRNAs acylated with d-α-Phe and (*R*)- and (*S*)-β-Phe monomers are disruptive to ternary complex formation and, thus, their utilization by the ribosome (Figures 2C,D and 4B,C). tRNAs acylated with d-α-Phe and (*R*)- and (*S*)-β^2^-Phe appeared unable to form a ternary complex with EF-Tu under the experimental conditions examined. Consequently, the incorporation of d-α-Phe- and (*R*)- and (*S*)-β^2^-Phe-tRNAs into the ribosome was below the detection limits of our tRNA selection smFRET assays. These results are consistent with previous studies that showed that d-α-Phe incorporation into dipeptides is 3 orders of magnitude slower compared to l-α-Phe incorporation.^15^ By contrast, (*R*)- and (*S*)-β^3^-Phe monomers inefficiently formed ternary complexes, reducing ternary complex abundance and thus the number of detectable tRNA selection events per unit time (Figure 4B,C). Strikingly, the proofreading stage of tRNA selection was accelerated for the events observed, consistent with higher rates of EF-Tu dissociation from both tRNA and the ribosome (Figure S5B,C). We infer from these findings that β^3^-Phe monomers lower ternary complex stability to an extent that the proofreading stage of tRNA selection after GTP hydrolysis is ostensibly bypassed. As l-α-amino acids misacylated onto non-native tRNAs have been reported to exhibit identical accommodation rates despite evidence of ternary complex formation defects,^71^ further experiments will be needed to discern whether the impact on proofreading is specific for β^3^-Phe monomers.

Our results also show that d-α-Phe and (*R*)-β^3^-Phe monomers exhibit significant reductions in the rate of downstream elongation reactions, including EF-G-catalyzed translocation (Figure 4E–G). (*R*)-β^3^-Phe, whose stereochemistry mimics that of an unnatural d-α-amino acid, showed significantly greater defects in ternary complex formation, tRNA accommodation during the proofreading stage of tRNA selection, and EF-G-catalyzed translocation than its (*S*)-β^3^-Phe counterpart. These observations are consistent with prior literature indicating that d-α-amino acids ((*R*)-α-amino acids) result in extended elongation pauses.^15,46,72^ In the cell, such pauses are likely accompanied by futile cycles of EF-Tu-catalyzed GTP hydrolysis^30^ and the induction of rescue pathways^73^ that may ultimately lead to compromises in cell growth and viability.

We conclude from these findings that ternary complex stability is a significant and perhaps underappreciated bottleneck that limits the incorporation of extended backbone monomers into polypeptides and proteins, both in vitro and in vivo. These observations warrant an examination of the extent to which ternary complex stability and EF-Tu-catalyzed tRNA selection defects impact the efficiency of nnAA utilization more broadly. Structural data^19,37,74,75^ suggest that the opposite chirality of d-α-Phe and the extended backbones of β^2^- and β^3^-Phe may introduce steric clashes at the interface with EF-Tu that alter its ability to both engage aminoacylated tRNA termini and undergo the rearrangements needed for stable ternary complex formation. Inspection and in silico analysis of the l-α-Phe-tRNA^Phe^ ternary complex suggests that d-α-Phe and all four β-Phe monomers sterically clash with EF-Tu, while *p*-Az-Phe does not (Figure S6). Reorientation of domains DI–III of EF-Tu, combined with repositioning of the aminoacylated tRNA termini, could, in principle, relieve the observed steric clashes with the β-Phe monomers, but this change would likely come at the expense of precise positioning of nucleotide binding pocket elements, including the S1 and S2 regions, and therefore ternary complex stability (Figure 1B). These findings support the hypothesis that engineering ternary complexes to compensate for nnAA-specific perturbations in stability could significantly improve incorporation efficiencies.

Further in-depth analyses of translation kinetics to address the issue of low non-α-amino acid utilization efficiency are warranted. To date, such studies have been performed under two different experimental approaches: in vitro translation^49,76^ or in cellulo incorporation.^69^ In vitro translation systems allow for the optimization of a wide range of experimental conditions and have been successful at incorporating diverse non-α-amino acid monomers in short peptides. However, their reaction yields are seldom reported, and only a minor fraction of published studies focus on relative rates or mechanistic bottlenecks.^16^ In cellulo studies that show the incorporation of nnAAs outside of α-amino or α-hydroxy acids are far fewer.^6−8,77^ An additional challenge is how to incorporate ribosome drop-off^78^ and quality control contributions^79^ into efficiency and yield calculations. Consequently, parallel in vitro and in cellulo investigations are likely to be the most informative.

Despite clear evidence that β-Phe monomers cause defects in ternary complex formation and stability, recent experiments show that β^3^-amino or β^2^-hydroxy acids can be incorporated into proteins in cells.^6,7,46^ β^3^-(*p*-Br)-Phe has also been incorporated into DHFR in *E. coli* cells expressing a ribosome containing a remodeled PTC.^6^ A β^2^-hydroxy analogue of N^ε^-Boc-l-α-lysine has also been incorporated into sfGFP by a wild-type *E. coli* strain,^7^ although the yield of protein was far lower than that expected from the in vitro activity of the aaRS. Kinetic simulations of ternary complex formation reveal that this discrepancy is likely explained by significant reductions in ternary complex concentration, due to lower stability, despite the presence of elevated EF-Tu concentrations in the cell (Figure S4). Perturbations to the tRNA selection mechanism, and proofreading specifically, are also expected to further lower the level of nnAA incorporation.

To achieve incorporation efficiencies approximating native amino acids, engineered EF-Tu and tRNA mutants and potentially other translation machinery components will likely be required to compensate for monomer-induced penalties to the system. Such efforts should aid the design of tailored orthogonal ternary complex components to complement existing genetic code expansion technologies, increasing nnAA tolerance and desired product yields.

## Experimental Procedures

### tRNA^Phe^ Purification and Fluorescent Labeling

Native tRNA^Phe^ was expressed and purified as described previously.^19,37,80^ Briefly, the pBS plasmid containing the tRNA^Phe^ V gene was transformed into JM109 cells and incubated overnight at 37 °C with shaking at 250 rpm. Cells were pelleted at 10 000*g* for 15 min. Then, cells were lysed by sonication in 20 mM potassium phosphate buffer (pH = 6.8) with 10 mM Mg(OAc)_2_ and 10 mM β-mercaptoethanol. Cellular debris was pelleted by high-speed centrifugation at 55 000*g* for 1.5 h. The supernatant was phenol:chloroform-extracted twice, followed by a series of precipitation steps consisting of an EtOH precipitation, two isopropanol precipitation steps (added dropwise), and a final EtOH precipitation. Bulk tRNA^Phe^ was then aminoacylated for 10 min in charging buffer (50 mM Tris-Cl, pH = 8.0; 20 mM KCl; 100 mM NH_4_Cl; 10 mM MgCl_2_; 1 mM DTT; 2.5 mM ATP; and 0.5 mM EDTA) with PheRS (crude tRNA in 45-fold molar excess) and a 10-fold molar excess of l-phenylalanine. Phe-tRNA^Phe^ was separated from crude tRNA using a TSK Phenyl-5PW hydrophobic interaction chromatography (HIC) column (Tosoh Bioscience) with a linear gradient starting from buffer A (10 mM NH_4_OAc, pH = 5.8; 1.7 M (NH_4_)_2_SO_4_) to buffer B (10 mM NH_4_OAc, pH = 5.8; 10% MeOH). Elution fractions corresponding to Phe-tRNA^Phe^ were pooled, dialyzed into storage buffer (10 mM KOAc, pH = 6.0; 1 mM MgCl_2_), and concentrated.

Phe-tRNA^Phe^ was fluorescently labeled with Cy3B Mono NHS ester (Cytiva) using the native aminocarboxypropyluridine (acp^3^) post-transcriptional modification at the U47 position with previously described procedures.^19,37^ Briefly, Phe-tRNA^Phe^ was buffer-exchanged into 312.5 mM HEPES at pH = 8.0. NaCl was added to a final concentration of 1 M. Then, 2 μL of 50 mM Cy3B in DMSO was added at 15 min intervals over a 1 h period. Reaction conditions were such that all of the acyl bond was hydrolyzed during labeling, yielding deacyl-tRNA^Phe^-Cy3B. Labeled tRNA^Phe^ was then phenol:chloroform-extracted, EtOH-precipitated, and purified over the TSK Phenyl-5PW column as described above. tRNA^Phe^-Cy3B was buffer-exchanged into storage buffer, concentrated, aliquoted, and stored at −80 °C until further use.

### Elongation Factor Purification and Fluorescent Labeling

His-tagged versions of elongation factors Tu and Ts were expressed from pPROEX vectors and purified by Ni^2+^-NTA, as described previously.^19,37^ Fluorescent EF-Tu was labeled on a C-terminal acyl carrier protein (ACP) tag with LD655-CoA via ACP synthase. Briefly, 5–10 mol equiv of LD655-CoA was mixed with EF-Tu-ACP in labeling buffer containing 50 mM HEPES (pH = 7.5) and 10 mM Mg(OAc)_2_. Labeled EF-Tu-LD655 was separated from ACP-S and free dye via Ni^2+^-NTA, and TEV protease was added to remove the 6X-His tag from EF-Tu-LD655 and run over a second Ni^2+^-NTA to remove TEV. EF-Tu-LD655/Ts complexes were purified by size exclusion chromatography, concentrated into factor storage buffer containing 10 mM HEPES (pH = 7.5), 100 mM KCl, 1 mM DTT, and 50% glycerol, and stored at −20 °C.

### Monomer Synthesis

The general procedure for l-α-Phe cyanomethyl ester (CME) monomer synthesis followed previously published procedures with slight modifications.^45^ To a 5 mL round-bottom flask, *N*-Boc-protected amino acid (0.5 mmol) was dissolved in 1 mL of tetrahydrofuran. The flask was then charged with 315 μL of chloroacetonitrile (5.0 mmol, 10 equiv), followed by the addition of 100 μL of *N*,*N*-diisopropylethylamine (0.6 mmol, 1.2 equiv). The flask was capped with septa and stirred at room temperature overnight, 16 h. The solvent was removed via rotary evaporation and then the crude material was purified by reverse-phase flash chromatography, 0–100% acetonitrile in water, holding at 60% acetonitrile until the product was collected. The solvent was removed via rotary evaporation, where the resulting oil was dissolved in 1 mL of tetrahydrofuran for deprotection. To the resulting solution, 1.9 mL of trifluoroacetic acid (25 mmol, 50 equiv) was added, and the mixture was left to be stirred at room temperature for 2 h. Upon completion, the solvent was removed, followed by purification by reverse-phase flash chromatography utilizing 2% acetonitrile in water mobile phase. The solvent was removed by lyophilization to yield the target materials.

The general procedure for β^3^-substituted phenylalanine CME monomers was performed as follows. The Boc-protected amino acid-CME (ca. 0.5 mmol) was treated with neat formic acid (2 mL). The solution was stirred at room temperature for 12 h before removing all of the formic acid under reduced pressure by azotropic distillation with CHCl_3_ to afford a pale yellow oil. The oil was dissolved in a minimum amount of THF (ca. 2 mL) and triturated with excess MTBE or Et_2_O until a white solid was formed persistently. All of the residual solvent was removed under reduced pressure. The white solid was crushed into fine powder, rinsed thoroughly with Et_2_O (10 mL), and dried under vacuum overnight. The typical yield over two steps was 50%.

### Flexizyme Charging of Native Cy3B-Labeled tRNA^Phe^

All concentrations listed are final; 5 mM of CME monomers was charged onto Cy3B-tRNA^Phe^ with a 5-fold excess of flexizyme. The flexizyme RNA oligo sequence^44,45,81^ used in this study is as follows: GGAUCGAAAGAUUUCCGCGGCCCCGAAAGGGGAUUAGCGUUAGGU. RNA oligos were ordered deprotected from IDT, resuspended in ultrapure water, flash frozen, and stored at −80 C. For l-α-Phe, d-α-Phe, and *p*-Az-Phe, charging reactions were carried out in 50 mM HEPES (pH = 6.6), 600 mM MgCl_2_, and 20% DMSO for 2 h (16 h for d-α-Phe) at 4 °C. For the β^2^-substituted Phe monomers, reactions were carried out in 50 mM bicine (pH = 9.0), 600 mM MgCl_2_, and 30% DMSO on ice for 24 h. For the β^3^-substituted Phe monomers, reactions were carried out in 50 mM bicine (pH = 9.0), 600 mM MgCl_2_, and 10% DMSO on ice for 24 h. All flexizyme charging reactions were quenched with 90 μL of 0.3 M NaOAc (pH = 5.3) and EtOH-precipitated at −20 °C. The precipitate was centrifuged at 21 000*g* for 10 min and EtOH-aspirated. Then, the precipitate was resuspended in buffer A, and the acylated species was purified from the deacylated species as described above for normal tRNA^Phe^ purification procedures. The purified, charged monomers were dialyzed into storage buffer and concentrated down via Amicon centrifugal filters with a 3K molecular weight cut off (MWCO). Samples were aliquoted, flash frozen, and stored at −80 °C. The extent of aminoacylation for each monomer-charged tRNA^Phe^ was verified immediately prior to use by HIC, which was performed at pH ∼ 6 to slow spontaneous deacylation during purification. To further reduce hydrolysis, aa-tRNA aliquots were transported in liquid nitrogen and thawed immediately before use.

### Ternary Complex Assay

Ternary complex assays were performed with a QuantaMaster-400 spectrofluorometer (Photon Technology International) with 520 and 570 nm excitation and emission wavelengths, respectively, and a 532 long-pass filter placed in front of the emission photomultiplier tube (PMT) to omit noise from excitation light. Rapid stopped-flow experiments were performed on a micro stopped-flow system (μSFM, BioLogic) equipped with a MOS-200/M spectrometer with the excitation monochromator set at 520 nm and a 582/75 bandpass filter in front of in the emission detector.^19,37^ In both cases, all concentrations listed are final. Ternary complex formation reactions were carried out in ternary complex reaction buffer containing 100 mM HEPES (pH = 7.4), 20 mM KCl, 100 mM NH_4_Cl, 1 mM DTT, 0.5 mM EDTA, and 2.5 mM Mg(OAc)_2_. Concentrated aliquots of aa-tRNA^Phe^-Cy3B were transferred in LN2, thawed, and diluted immediately before use to prevent hydrolysis of the aminoacyl moiety. Briefly, ternary complex formation was achieved by stopped-flow injection of 400 nM (unless specified otherwise) EF-Tu-LD655 into a solution containing 5 nM aa-tRNA^Phe^-Cy3B in a ternary complex reaction buffer. Prior to stopped-flow injection, EF-Tu-LD655 was pre-incubated in ternary complex reaction buffer with 10 μM GTP with or without EF-Ts, as indicated. Upon reaction equilibration, ternary complex dissociation was achieved by the stopped-flow injection of 100 μM GDP in ternary complex buffer to the same solution. For the μSFM system, equal volumes of EF-Tu-LD655 with or without 3 μM EF-Ts and aa-tRNA^Phe^-Cy3B were rapidly mixed together (final volume of 24 μL, flow rate of 1.2 mL/s). The formation reaction was monitored at 800 V with sampling times of 1 ms for the first 5 s and 10 ms for the remaining reaction time. All relative fluorescence values were plotted against time and fit to
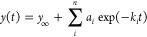
where *n* = 1 or 2, as required.

### Kinetic Simulations

Kinetic analysis of the ternary complex formation assays was performed in MATLAB (2021b). *k*_on_ and *k*_off_ values derived from the stopped-flow data were used in combination with previously reported values to simulate ternary complex levels at physiological concentrations. A system of ordinary differential equations based on the kinetic model in Figure S4A was solved using the function ode89 in MATLAB at different combinations of aa-tRNA, EF-Tu, and EF-Ts concentrations. A proportion of 4 × [EF-Tu] = [EF-Ts] was kept constant for each aa-tRNA-EF-Tu pair. Previously reported *E. coli* cytoplasmic concentrations of GDP (0.69 mM) and GTP (4.9 mM) were used. The equilibrium concentrations of aa-tRNA free and bound were used to determine the ternary complex fraction with the following equation:
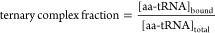


### Single-Molecule FRET Experiments

Single-molecule FRET experiments were performed by using a custom-built prism-type TIRF microscope. Bacterial ribosomes programed with Cy3-fMet-tRNA^fMet^ in the P site and a UUC codon displayed in the A site were surface immobilized via a streptavidin–biotin interaction to a transparent surface passivated with PEG polymers doped with biotin-PEG. tRNA selection experiments were initiated by the injection of preformed ternary complexes (aa-tRNA-LD655, 12.5 nM; EF-Tu/Ts, 125 nM; and GTP or GTPγS, 500 μM) in bacterial polymix buffer (50 mM Tris-OAc (pH = 7.5), 100 mM KCl, 5 mM NH_4_OAc, 0.5 mM Ca(OAc)_2_, 5 mM Mg(OAc)_2_, 6 mM 2-mercaptoethanol, 0.1 mM EDTA, 5 mM putrescine, and 1 mM spermidine) supplemented with 2 mM PCA/PCD oxygen scavenging system and 1 mM each of cyclooctatetraene (COT), nitrobenzyl alcohol (NBA), and Trolox triplet state quenchers. Translocation experiments were initiated by the injection of preformed ternary complexes (aa-tRNA-LD655, 25 nM; EF-Tu/Ts, 250 nM; and GTP, 1250 μM) supplemented with 8 μM EF-G in bacterial polymix buffer. Samples were illuminated with a 532 nm diode pumped solid-state laser (Opus, LaserQuantum) at 0.4 and 0.05 kW cm^–2^ with 10 or 100 ms integration times, respectively. Equilibrium movies of pretranslocation complexes were acquired at 0.12 kW cm^–2^ with a 40 ms integration time. Full elongation cycle movies were acquired at 0.01 kW cm^–2^ with a 200 ms integration time. Fluorescence emission from donor and acceptor fluorophores was collected using a 60×/1.27 NA super-resolution water-immersion objective (Nikon). Fluorescence was recorded onto two aligned ORCA-Fusion sCMOS cameras (C-14440-20UP, Hamamatsu). Instrument control was performed using custom software written in LabVIEW (National Instruments). Fluorescence intensities were extracted from the recorded videos, and FRET efficiency traces were calculated using the SPARTAN software package. FRET traces were selected for further analysis according to the following criteria: 8:1 signal/background noise ratio and 6:1 signal/signal noise ratio, less than four donor-fluorophore blinking events, and a correlation coefficient between donor and acceptor of <0.5. The resulting smFRET traces were further post-synchronized to the appearance of FRET and analyzed using the segmental *k-*means (SKM) algorithm, as implemented in the SPARTAN software package v3.8. Data were plotted in OriginPro 2019b (OriginLab, Northampton, Massachusetts, U.S.). Dwell time curves were fit to
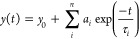
where *n* = 1 or 3, as required. Mean and standard deviations (SD) of the classical–hybrid split ratio were calculated from four independent measurements. Statistical significance (*p* < 0.05) was assessed by a two-way analysis of variance (ANOVA) followed by a post hoc Bonferroni test. Apparent rates of ternary complex arrival were estimated by first constructing, per injection, a cumulative distribution of the dwell times from the ternary complex injection time to the first evidence of FRET ≥0.2. Distributions were fit to a sum of two exponentials (see above) with a delay to account for the mixing time. Error bars denote standard deviation from *n* = 5–8 injections. GA-state lifetimes were estimated by collecting dwell times from HMM idealizations into survival plots, which were fit to a sum of two exponentials (see above). Reported lifetimes are taken as the time constant of the exponential component with the highest (53–98%) amplitude. Error bars denote the standard error calculated from 1000 bootstrap resamples.

## Supporting Information Available

Figures S1–S6 and synthesis of Boc-protected β^2^-Phe amino acids and α-amino acid-cyanomethyl esters (PDF)
