# Supplementary material for: β-Amino Acids Reduce Ternary Complex Stability and Alter the Translation Elongation Mechanism
Source: ACS Cent Sci. 2024 Jun 4;10(6):1262–75. doi: 10.1021/acscentsci.4c00314 (PMC11212133; doi:10.1021/acscentsci.4c00314)
Supplement: Supplementary file 1 — oc4c00314_si_001.pdf [file oc4c00314_si_001.pdf]

β-amino acids reduce ternary complex stability and alter the translation elongation mechanism

F. Aaron Cruz-Navarrete[1,2,#], Wezley C. Griffin[1,2,#], Yuk-Cheung Chan[3,#], Maxwell I. Martin[1,2], Jose L. Alejo[1,2], Ryan A. Brady[1,2], S. Kundhavai Natchiar[1,2], Isaac J. Knudson[4], Roger B. Altman[1,2], Alanna Schepartz[4,5,6,7,8], Scott J. Miller[3,*], and Scott C. Blanchard[1,2,*]

[1]Department of Structural Biology, St Jude Children's Research Hospital, Memphis, Tennessee, USA

[2]Department of Chemical Biology & Therapeutics, St Jude Children's Research Hospital, Memphis, Tennessee, USA

[3]Department of Chemistry, Yale University, New Haven, Connecticut, USA

[4]College of Chemistry, University of California, Berkeley, California, USA

[5]Molecular and Cell Biology, University of California, Berkeley, CA 94720, USA

[6]California Institute for Quantitative Biosciences, University of California, Berkeley, CA 94720, USA

[7]Chan Zuckerberg Biohub, San Francisco, CA 94158, USA

[8]Innovation Investigator, ARC Institute, Palo Alto, CA 94304, USA

[#]Authors contributed equally.

*Email: scott.miller@yale.edu; scott.blanchard@stjude.org

Table of Contents

Figure S1. Aminoacylation analysis of various aa-tRNA^{Phe}-Cy3B……………………………….…2

Figure S2. Ternary complex formation assay comparing Flexizyme and PheRS-charged L-α-Phe-tRNA^{Phe}-Cy3B……………………………………………………………………………..……4

Figure S3. Ensemble FRET assay using mutant tRNA^{Phe} (C49A G65U) and/or EF-Tu (N273A)…………………………………………………………………………………...……5

Figure S4. Kinetic simulations of ternary complex formation……………………………….…….7

Figure S5. Quantitative kinetic analysis of high time-resolution smFRET data………..……….9

Figure S6. Manual modelling of aa-tRNA^{Phe} into the EF-Tu binding pocket……………….……10

Synthesis of Boc-Protected β^2-Phe amino acids……………………………………………..11

Synthesis of α-amino acid-cyanomethyl esters………………………………………………..23

Figure S1

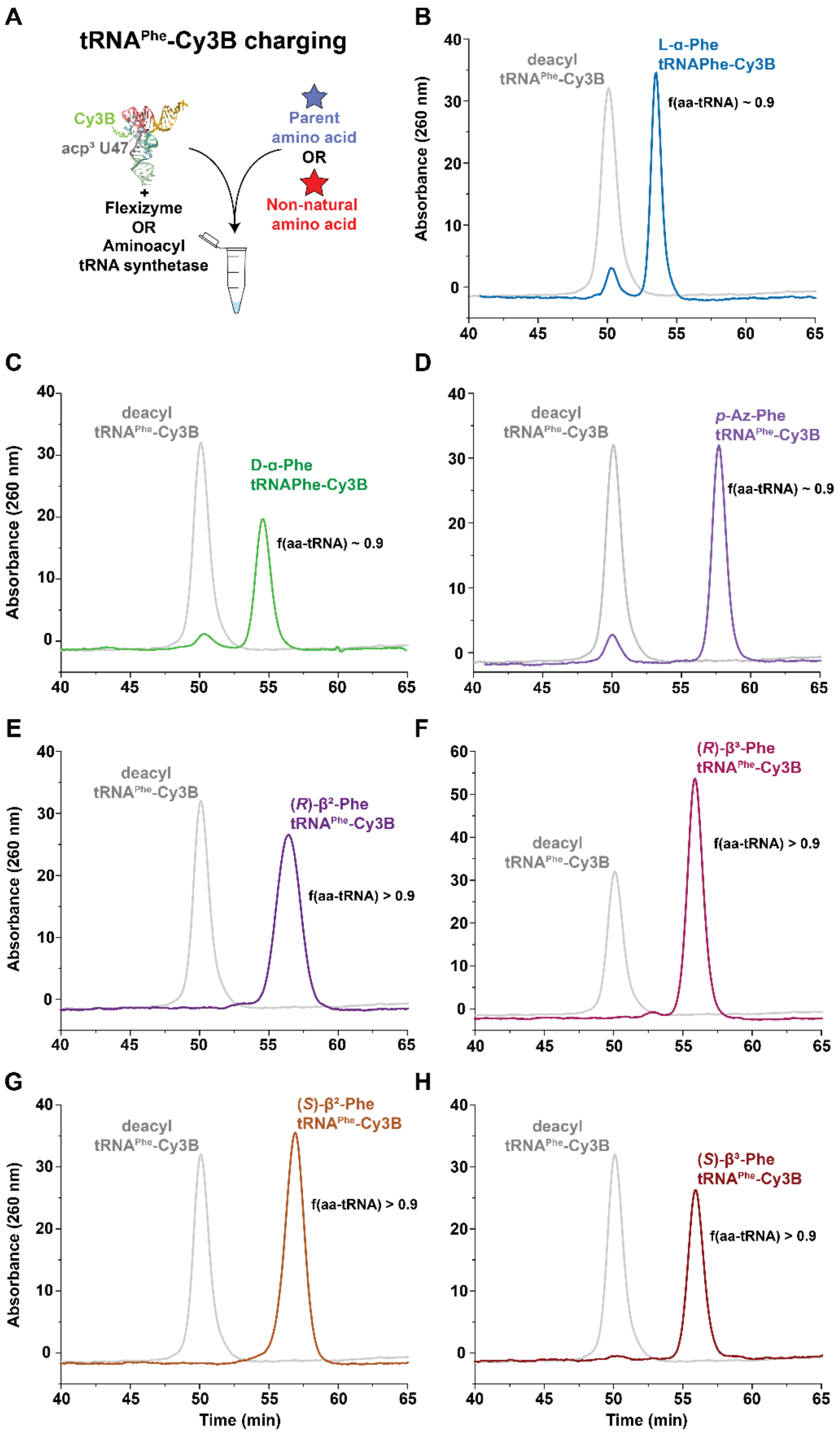
A
tRNAPhe-Cy3B charging
Cy3B
acp³ U47
+
Flexizyme
OR
Aminoacyl
tRNA synthetase
Parent
amino acid
OR
Non-natural
amino acid
B
deacyl
tRNAPhe-Cy3B
L-α-Phe
tRNAPhe-Cy3B
f(aa-tRNA) ~ 0.9
Absorbance (260 nm)
C
deacyl
tRNAPhe-Cy3B
D-α-Phe
tRNAPhe-Cy3B
f(aa-tRNA) ~ 0.9
D
deacyl
tRNAPhe-Cy3B
p-Az-Phe
tRNAPhe-Cy3B
f(aa-tRNA) ~ 0.9
E
deacyl
tRNAPhe-Cy3B
(R)-β²-Phe
tRNAPhe-Cy3B
f(aa-tRNA) > 0.9
F
(R)-β³-Phe
tRNAPhe-Cy3B
deacyl
tRNAPhe-Cy3B
f(aa-tRNA) > 0.9
G
deacyl
tRNAPhe-Cy3B
(S)-β²-Phe
tRNAPhe-Cy3B
f(aa-tRNA) > 0.9
H
deacyl
tRNAPhe-Cy3B
(S)-β³-Phe
tRNAPhe-Cy3B
f(aa-tRNA) > 0.9
Time (min)

Figure S1. Verification of the extent of aminoacylated $tRNA^{Phe}$ in stored aa-$tRNA^{Phe}$ stocks. A) Cartoon schematic of $tRNA^{Phe}$-Cy3B charging. $tRNA^{Phe}$-Cy3B is aminoacylated by mixing with an excess of flexizyme or tRNA synthetase and the specified parent (blue) or non-natural (red) amino acid (**Experimental Procedures**). Extent of aminoacylated $tRNA^{Phe}$ in stored stock aliquots for **B)** L-α-Phe, **C**) D-α-Phe, **D**) *p*-Az-Phe, **E**) (*R*)-β^2-Phe, **F**) (*R*)-β^3-Phe, **G**) (*S*)-β^2-Phe, **H**) (*S*)-β^3-Phe. Fraction of aa-tRNA shown in each graph. Chromatogram of deacylated $tRNA^{Phe}$-Cy3B (grey) is included for comparison.

Figure S2

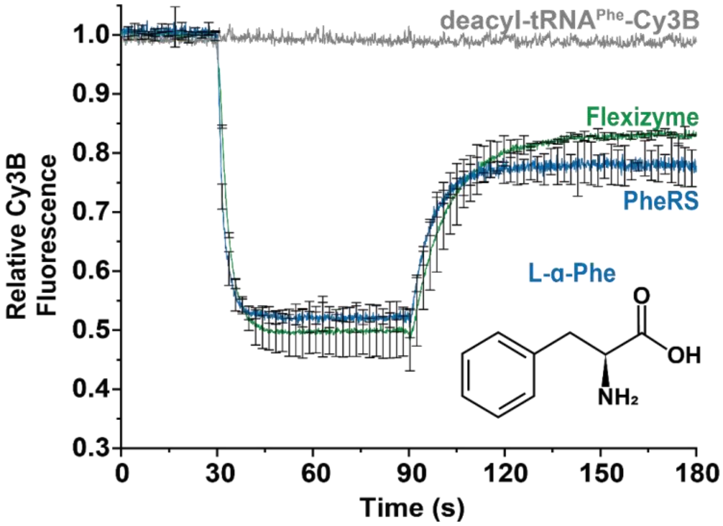

Figure S2. Ternary complex formation assay comparing Flexizyme and PheRS-charged L-α-Phe-tRNA^{Phe}-Cy3B. Ternary complex formation assay as described in Figure 2 comparing tRNA^{Phe}-Cy3B charged by PheRS (blue) or flexizyme (green), and de-acylated tRNA^{Phe}-Cy3B (grey). Error bars represent S.D. from two separate replicates.

Figure S3

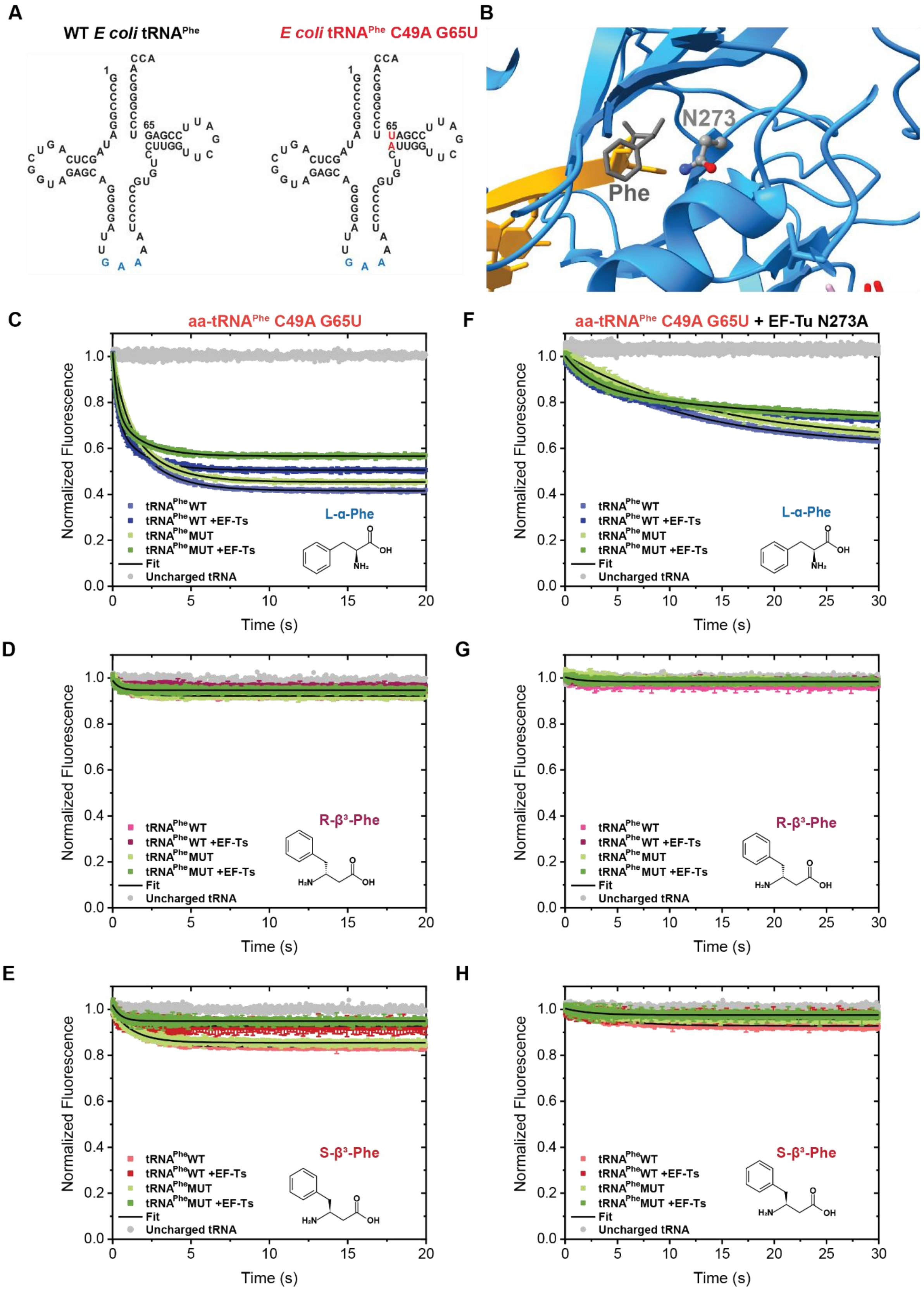

A
WT E coli tRNAPhe
E coli tRNAPhe C49A G65U
B
N273
Phe
C
aa-tRNAPhe C49A G65U
F
aa-tRNAPhe C49A G65U + EF-Tu N273A
Normalized Fluorescence
Time (s)
tRNAPhe WT
tRNAPhe WT +EF-Ts
tRNAPhe MUT
tRNAPhe MUT +EF-Ts
Fit
Uncharged tRNA
L-α-Phe
D
G
R-β³-Phe
E
H
S-β³-Phe

Figure S3. Ensemble FRET assay using mutant tRNA^{Phe} (C49A G65U) and/or EF-Tu (N273A). A) Sequences of WT (left) and mutant (right) *E coli* tRNA^{Phe} used for stopped-flow kinetic experiments. **B)** Structure of EF-Tu (PDB: 1OB2) with the N273 amino acid residue in the amino acid binding pocket shown next to the Phe (gray) aminoacylated to the A76 on the 3'-end of tRNA^{Phe} (yellow). **C-E)** Stopped-flow ternary complex formation assays using Cy3B-labeled mutant aa-tRNA^{Phe} (MUT) with the indicated monomers inset. **F-H)** Stopped-flow ternary complex assays with both tRNA^{Phe} MUT and mutant EF-Tu N273A with the indicated monomers inset. Error bars represent S.D. of 3-5 replicates.

Figure S4

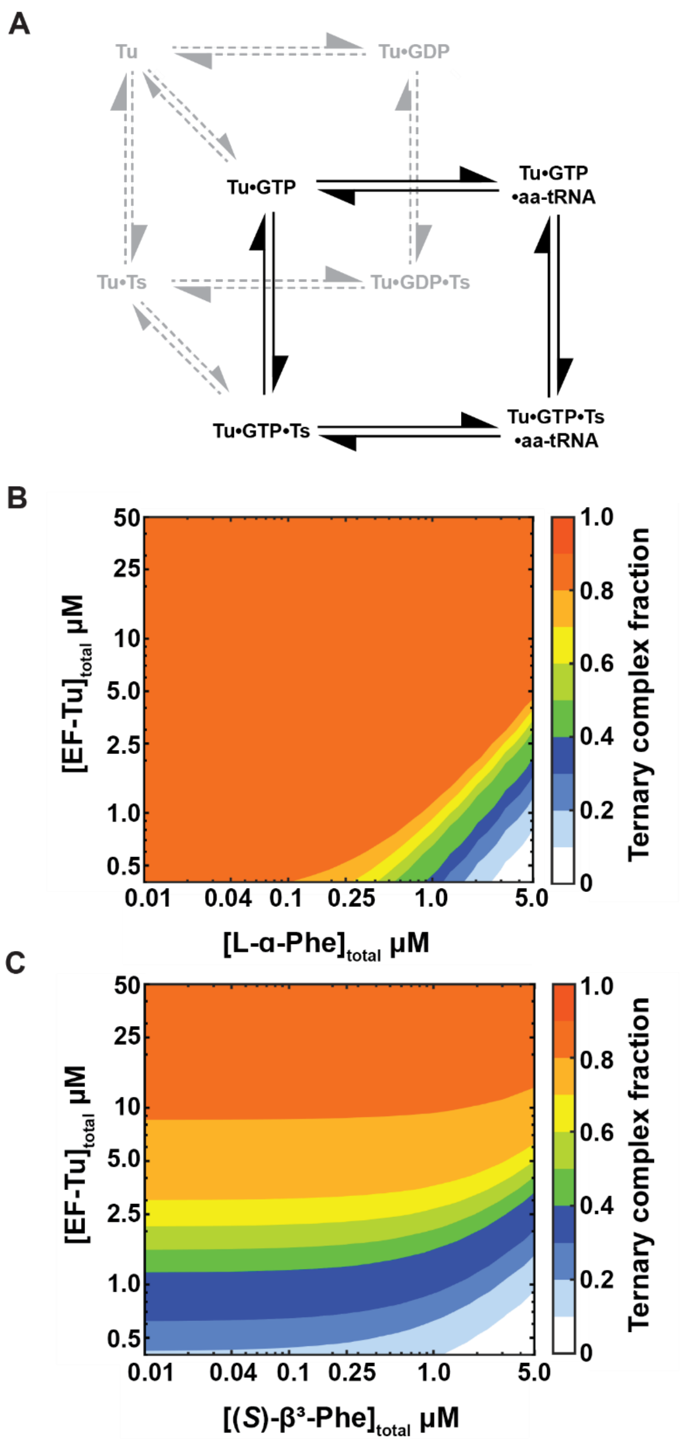
A
Tu
Tu•GDP
Tu•GTP
Tu•GTP
•aa-tRNA
Tu•Ts
Tu•GDP•Ts
Tu•GTP•Ts
Tu•GTP•Ts
•aa-tRNA
B
[EF-Tu]total μM
50
25
10
5.0
2.5
1.0
0.5
0.01
0.04
0.1
0.25
1.0
5.0
[L-α-Phe]total μM
1.0
0.8
0.6
0.4
0.2
0
Ternary complex fraction
C
[EF-Tu]total μM
50
25
10
5.0
2.5
1.0
0.5
0.01
0.04
0.1
0.25
1.0
5.0
[(S)-β³-Phe]total μM
1.0
0.8
0.6
0.4
0.2
0
Ternary complex fraction

Figure S4. Kinetic simulations of ternary complex formation. A) Minimal reaction scheme for kinetic simulations. Contour plots of **B)** L-α-Phe and **C)** (*S*)-β^3-Phe ternary complex abundance simulated at different physiologically relevant aa-tRNA and ET-Tu/Ts concentrations. Simulations were done using measured rate constants from experiments reported here and from the literature. Ternary complex fraction was calculated as $[\text{aa-tRNA}]_{bound}/[\text{aa-tRNA}]_{total}$

Figure S5

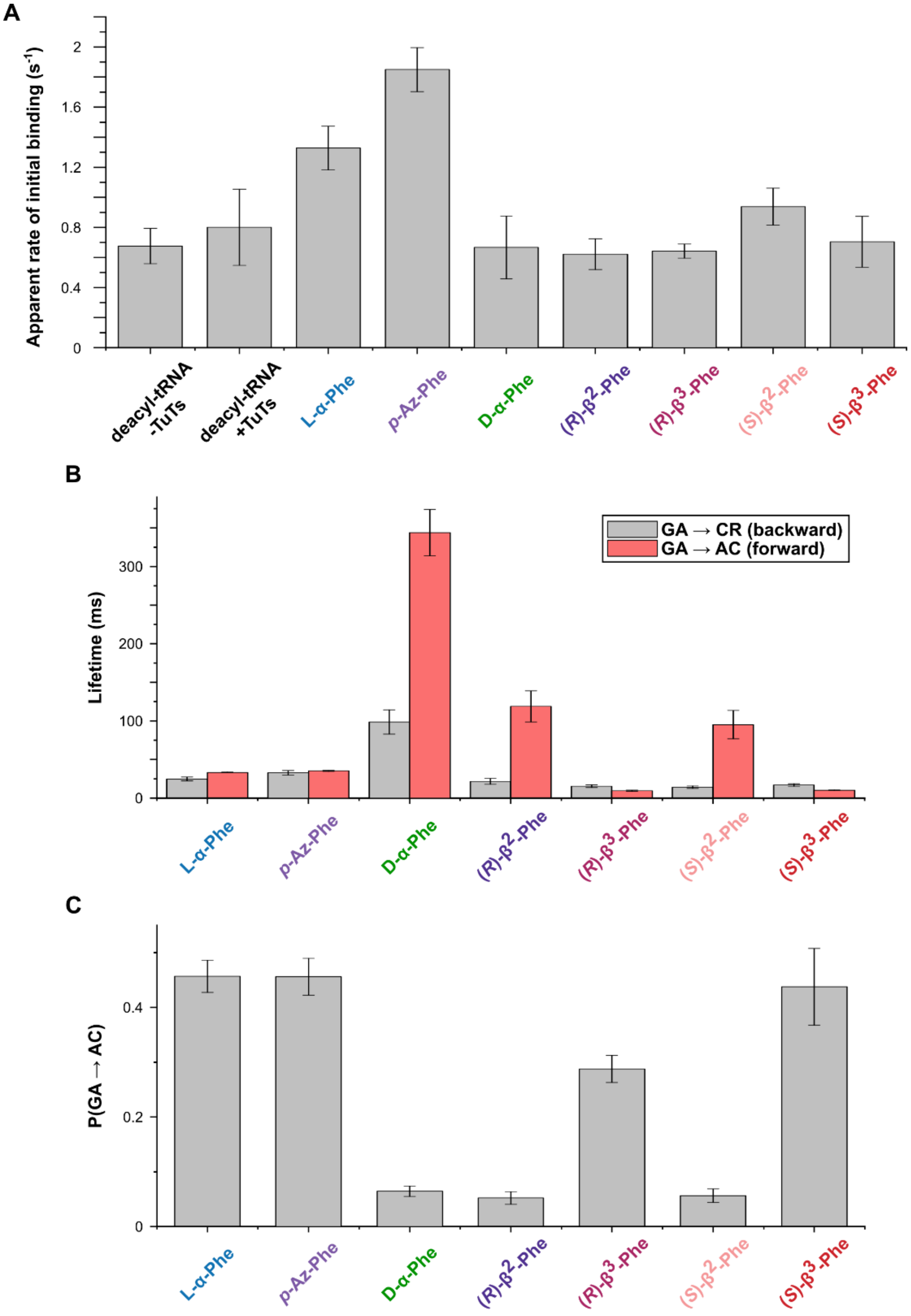

Figure S5. Quantitative kinetic analysis of high time-resolution smFRET data. **A**) Apparent initial binding rate determined from the time between injection of ternary complex to first FRET ≥ 0.2. **B**) Forward (GA → AC) and backward (GA → CR) lifetimes of rate-limiting step of tRNA selection from the GTPase-activated state to full accommodation. **C**) Probability of accommodating from the GA state, as determined by the branching ratio exiting the GA state calculated from HMM idealized smFRET trajectories.

Figure S6

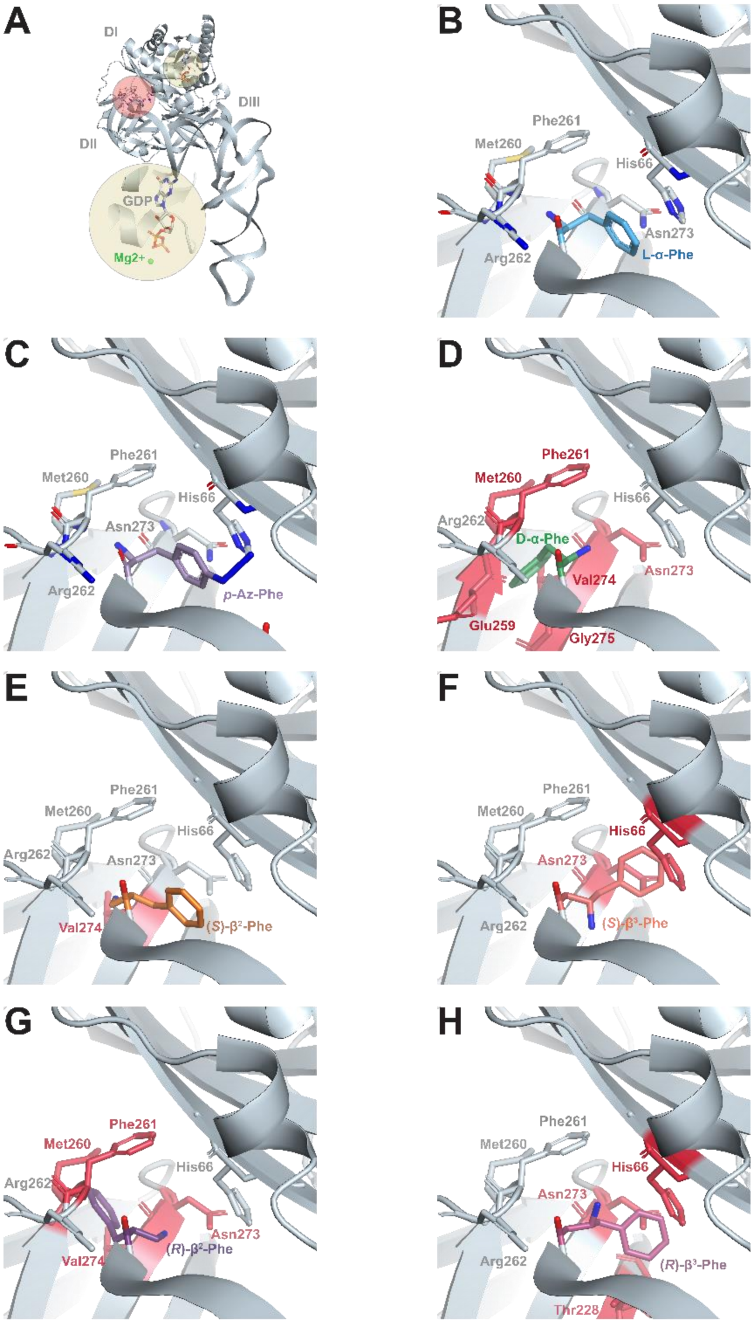

Figure S6. Manual modelling of aa-tRNA^{Phe} into the EF-Tu binding pocket. A) Ternary complex structure used to model all aa-tRNA^{Phe} studied. EF-Tu nucleotide binding pocket containing GDP and Mg^{2+} is circled in yellow and zoomed in. The amino acid binging pocket is circled in red. Manual modelling of **B)** L-α-Phe, **C)** *p*-Az-Phe, **D)** D-α-Phe **E)** (*S*)-β^2-Phe, **F)** (*S*)-β^3-Phe, **G)** (*R*)-β^2-Phe, **H)** (*R*)-β^3-Phe into the amino acid binding pocket of EF-Tu formed by the DI-DII interface. Steric clashes are represented in red. The 2D model of non-natural amino acids was created using the Chemical Sketch Tool provided by the RCSB Protein Data Bank. Subsequently, the 3D atomic models and RNA-peptide links were constructed using JLigand [80].

The figures were generated using PYMOL, the PyMOL Molecular Graphics System, Version 2.5.7 Schrödinger, LLC.

Synthesis of Boc-Protected β^2-amino acids.

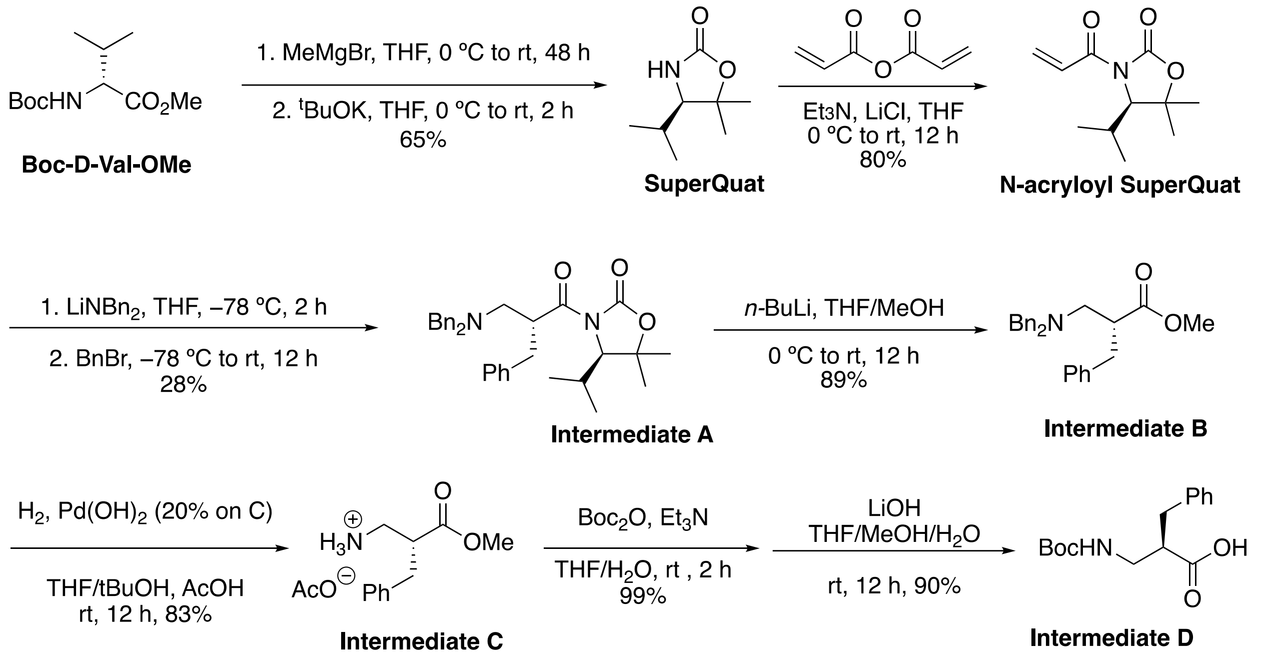

Note: The enantiomer of **Intermediate D** was prepared from Boc-L-Val-OMe.

SuperQuat and **N-acryloyl SuperQuat** were prepared according to literature procedure (Asymmetric synthesis of b2-amino acids: 2-substituted-3-aminopropanoic acids from N-acryloyl SuperQuat derivatives. *Org. Biomol. Chem*., **2007**, *5*, 2812–2825.) **Intermediate A** and **B** were also prepared according to the same literature. For **Intermediate A**, we used a different eluent system (hexanes:EtOAc 10 :1 to 7:1, instead of hexanes:Et_2O 20:1 in the literature condition) for the SiO_2 column chromatographic purification to afford **Intermediate A** as pale yellow oil (980 mg, 1.96 mmol) in 28% yield (from 7 mmol **N-acryloyl SuperQuat**). For **Intermediate B**, same literature procedure was followed to afford **Intermediate B** as colorless oil (650 mg,1.74 mmol) in 89% yield (from 1.96 mmol of **A**).

Note: The enantiomer of **Intermediate A** and **B** were prepared from Boc-L-Val-OMe as the starting material by the same procedure.

Intermediate A

(*R*)-3-((*R*)-2-benzyl-3-(dibenzylamino)propanoyl)-4-isopropyl-5,5-dimethyloxazolidin-2-one

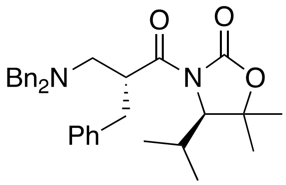

^{1}H NMR (400 MHz, CD_2Cl_2) δ 7.42 – 7.26 (m, 15H), 4.94 (tt, *J* = 8.6, 6.0 Hz, 1H), 4.23 (d, *J* = 2.8 Hz, 1H), 3.73 (d, *J* = 13.6 Hz, 2H), 3.65 (d, *J* = 13.7 Hz, 2H), 3.11 – 2.89 (m, 3H), 2.59 (dd, *J* = 12.6, 5.6 Hz, 1H), 2.05 (pd, *J* = 6.9, 2.8 Hz, 1H), 1.53 (d, *J* = 3.0 Hz, 6H), 0.83 (d, *J* = 7.0 Hz, 3H), 0.69 (d, *J* = 6.8 Hz, 3H).
^{13}C NMR (101 MHz, CD_2Cl_2) δ 175.6, 153.8, 139.6, 139.4, 129.7, 129.6, 128.8, 128.5, 127.3, 126.7, 82.7, 66.7, 58.9, 56.6, 43.1, 37.6, 29.9, 29.1, 21.5, 21.4, 16.6.

***enantiomer*-Intermediate A**

(*S*)-3-((*S*)-2-benzyl-3-(dibenzylamino)propanoyl)-4-isopropyl-5,5-dimethyloxazolidin-2-one

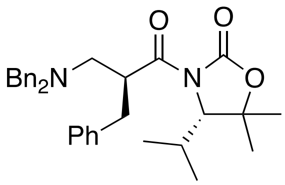

^{1}H NMR (500 MHz, CD_2Cl_2) δ 7.39 – 7.18 (m, 15H), 4.84 (tt, *J* = 8.5, 6.0 Hz, 1H), 4.16 (d, *J* = 2.8 Hz, 1H), 3.65 (d, *J* = 13.6 Hz, 2H), 3.58 (d, *J* = 13.6 Hz, 2H), 3.02 – 2.90 (m, 2H), 2.85 (dd, *J* = 13.6, 8.8 Hz, 1H), 2.51 (dd, *J* = 12.6, 5.7 Hz, 1H), 2.06 – 1.95 (m, 1H), 1.49 (d, *J* = 13.8 Hz, 6H), 0.77 (d, *J* = 7.0 Hz, 3H), 0.63 (d, *J* = 6.8 Hz, 3H).
^{13}C NMR (126 MHz, CD_2Cl_2) δ 175.6, 153.8, 139.6, 139.5, 129.6, 129.5, 128.7, 128.5, 127.3, 126.6, 82.8, 66.8, 58.9, 56.5, 43.1, 37.6, 29.9, 29.1, 21.5, 21.3, 16.6.

For **Intermediate B** to **Intermediate C**, we modified the condition (Literature: *Org. Biomol. Chem*., **2007**, *5*, 2812–2825.) from Pd/C in MeOH/AcOH into $Pd(OH)_2$/C (10% by weight w.r.t. substrate) in THF/*t*-BuOH (v:v, 3:1, 0.1 M) with AcOH (10% by volume) as additive.

Intermediate B (650 mg, 1.74 mmol) was dissolved in THF/*t*-BuOH (v:v, 3:1, total 16 mL, *ca*. 0.1 M) and AcOH (1.6 mL). $Pd(OH)_2$ (20% on carbon) (65 mg) was added to the solution. The reaction was degassed, connected to a H_2 balloon and stirred at rt for overnight before filtration over Celite. The filtrate was concentrated under reduced pressure to afford **Intermediate C** as oil (364 mg, 1.44 mmol, 83%) which was subjected to next step without further purification. Same experimental sequence was carried out to prepare the enantiomer of **Intermediate C**.

For **Intermediate C** to **Intermediate D**, **C** (364 mg, *ca*. 1.44 mmol, 1 equiv.) was dissolved in THF/H_2O (6 mL:2 mL). Et_3N (0.6 mL, 4.3 mmol, 3 equiv.) and Boc_2O (377 mg, 1.7 mmol, 1.2 equiv.) were added. The solution was stirred at rt for 2 h before dilution with EtOAc (10 mL). The organic phase was washed successively with 10% citric acid solution, saturated $NaHCO_3$ solution

and brine. The organic phase was dried over anhydrous Na_2SO_4, filtered and concentrated to afford (*R*)-Boc-β2-Phe-OMe (423 mg, 1.44 mmol), which was subjected to next step without further purification. LiOH•H_2O (242 mg, 7.2 mmol, 4 equiv.) and THF/MeOH/H_2O (4 mL/1 mL/1 mL) were added to the crude product. The mixture was stirred at rt for overnight before dilution with 4 mL H_2O. The aqueous phase was washed against Et_2O (5 mL × 2), followed by acidifying with 1N HCl to adjust the pH into ~2. The aqueous phase was extracted with EtOAc (15 mL × 2), dried over anhydrous Na_2SO_4, filtered and concentrated under reduced pressure to afford **Intermediate D** (363 mg, 1.3 mmol, 90%) as a colorless oil. The same experimental sequence was carried out to prepare the enantiomer of **Intermediate D**.

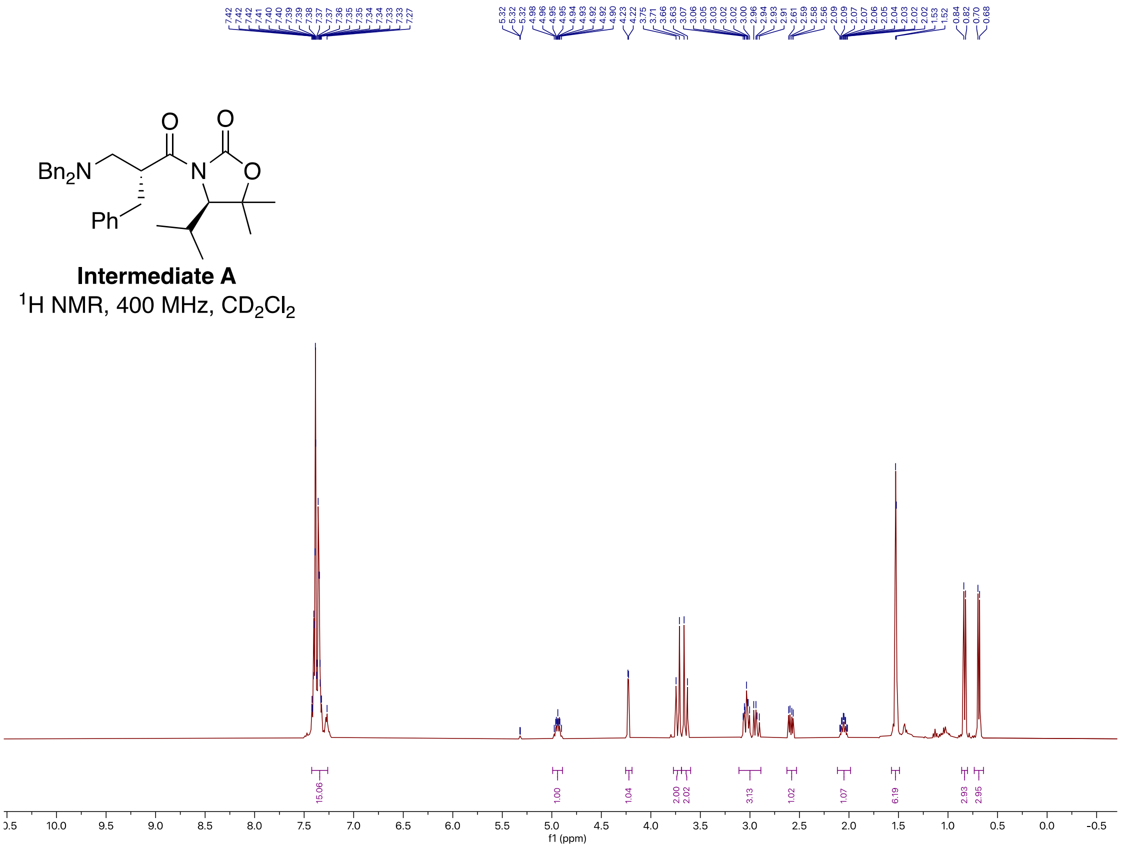
7.42 7.42 7.41 7.40 7.40 7.39 7.39 7.38 7.37 7.37 7.37 7.36 7.35 7.35 7.34 7.34 7.33 7.33 7.27
5.32 5.32 5.32 4.98 4.96 4.95 4.95 4.94 4.93 4.92 4.92 4.92 4.90 4.23 4.22 3.75 3.71 3.66 3.63 3.07 3.06 3.05 3.03 3.02 3.02 3.00 2.96 2.94 2.93 2.91 2.61 2.59 2.58 2.56 2.09 2.09 2.07 2.07 2.06 2.05 2.04 2.03 2.02 2.02 1.53 1.52 0.84 0.82 0.70 0.68
Bn2N
Ph
O
O
N
O
Intermediate A
1H NMR, 400 MHz, CD2Cl2
15.06
1.00
1.04
2.00
2.02
3.13
1.02
1.07
6.19
2.93
2.95
f1 (ppm)

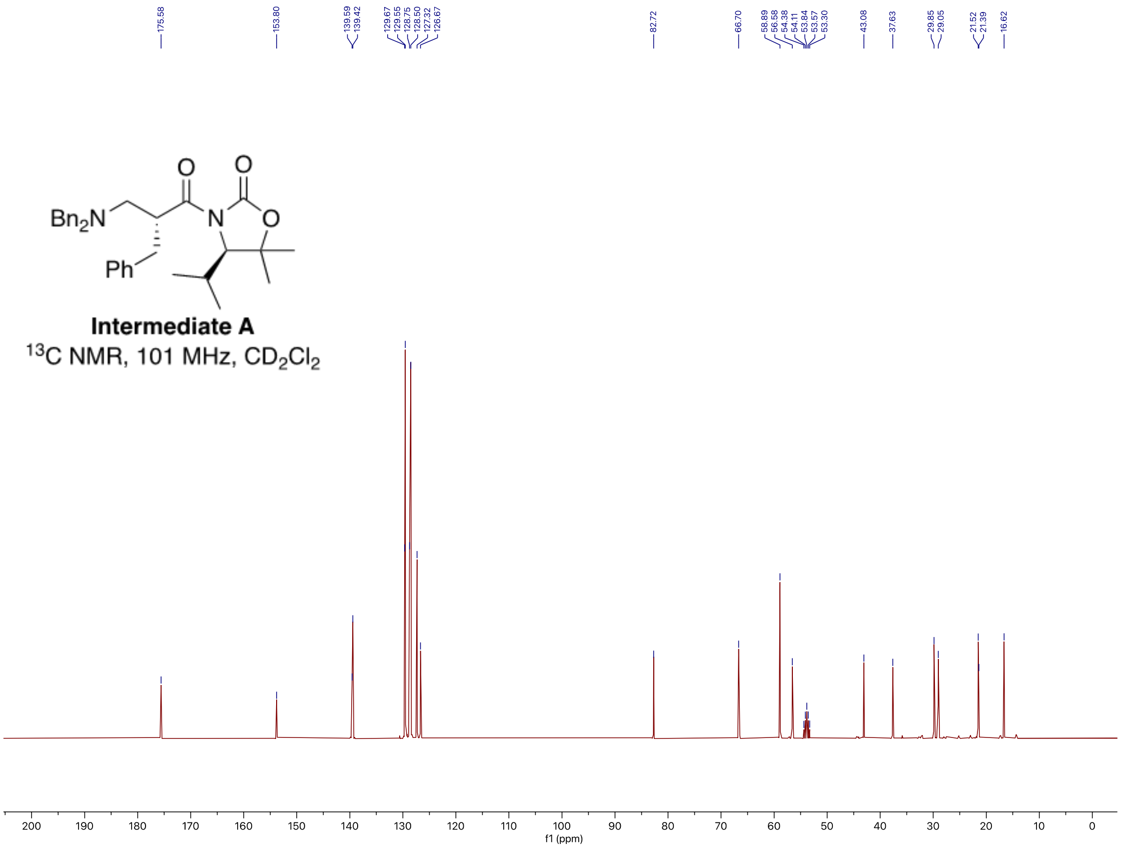
175.58
153.80
139.59 139.42
129.67 129.55 128.75 128.50 127.32 126.67
82.72
66.70
58.89 56.58 54.38 54.11 53.84 53.57 53.30
43.08
37.63
29.85 29.05
21.52 21.39
16.62
Bn2N
Ph
O
O
N
O
Intermediate A
13C NMR, 101 MHz, CD2Cl2
f1 (ppm)

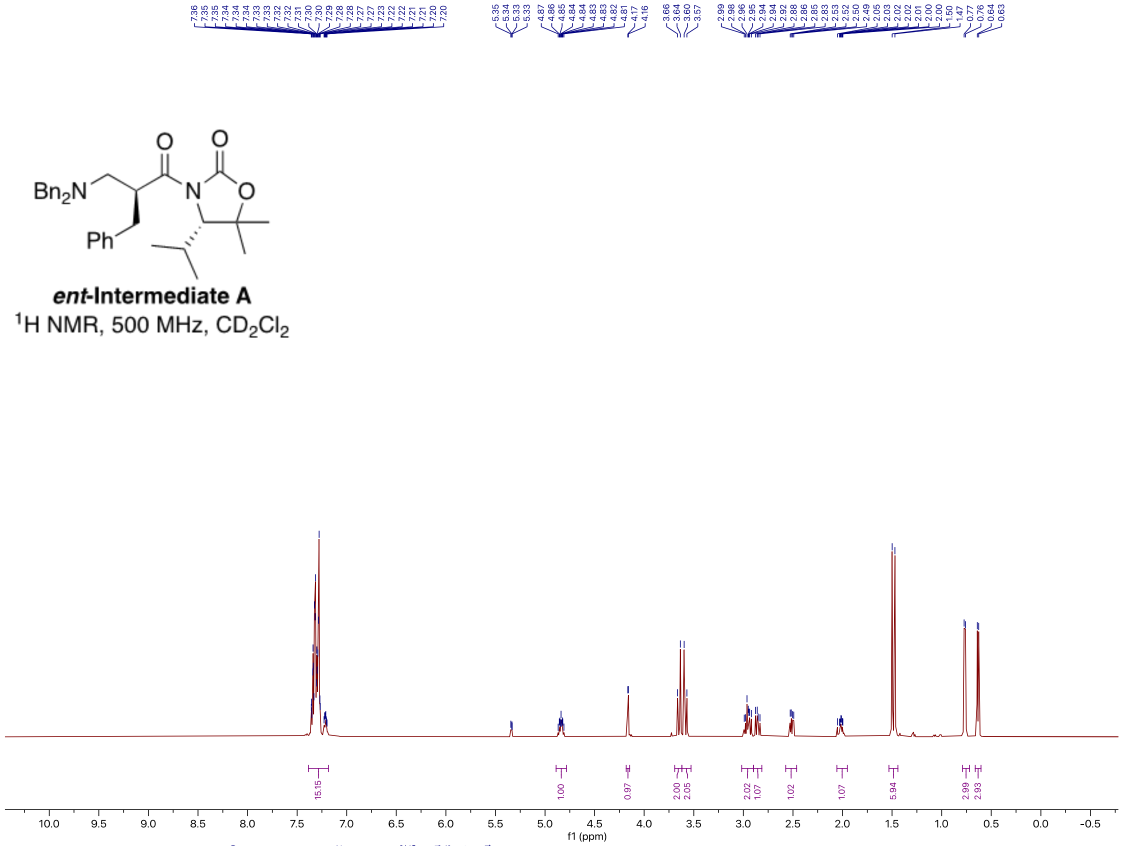

***ent*-Intermediate A**

^{1}H NMR, 500 MHz, CD_2Cl_2

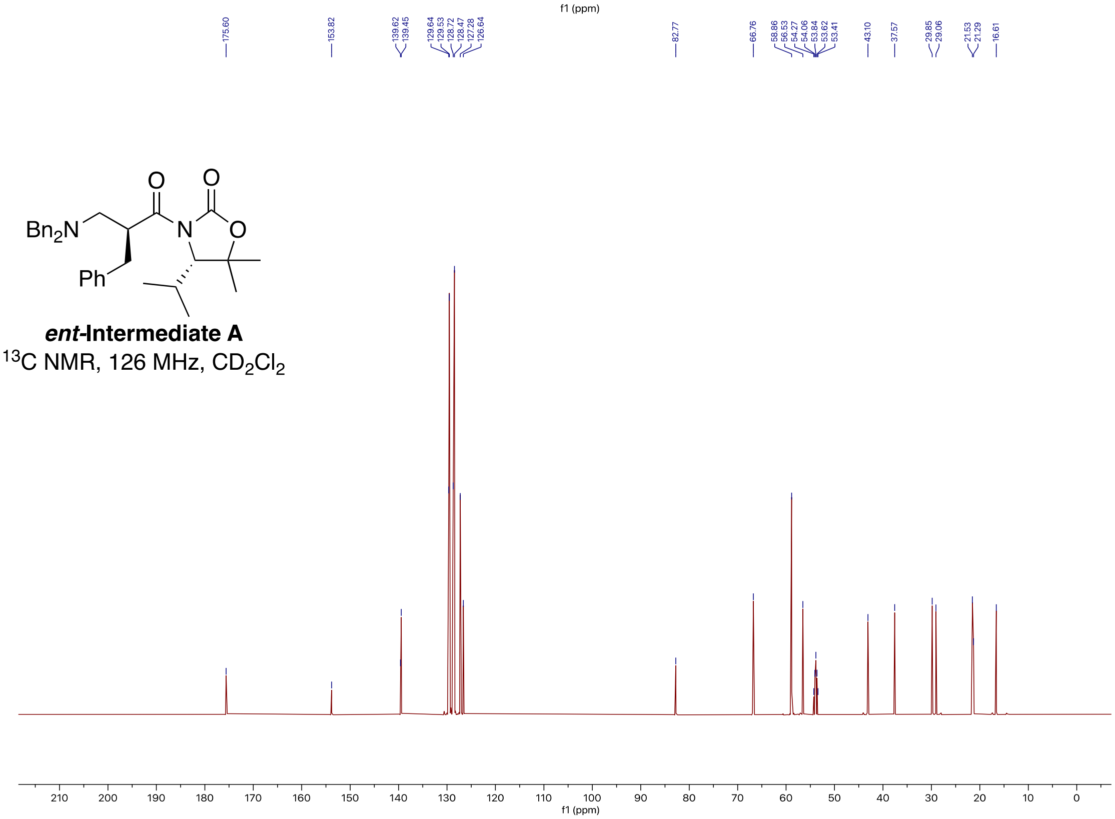

***ent*-Intermediate A**

^{13}C NMR, 126 MHz, CD_2Cl_2

General procedure for synthesis of β-amino acid-cyanomethyl ester-formate salt

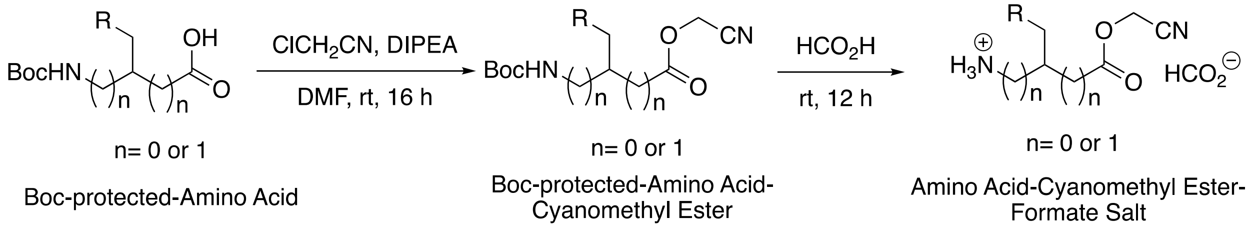

To a stirred solution of Boc-protected-amino acid (0.5 mmol, 1 equiv.) was added dry DMF (1 mL), $ClCH_2CN$ (0.75 mmol, 1.5 equiv.) and dry DIPEA (1 mmol, 2 equiv.), the solution was stirred at rt for 16 h before dilution with EtOAc (10 mL). The organic phase was washed successively with 10% citric acid aq. solution, 5 % LiCl aq. solution and brine. The organic phase was dried over anhydrous Na_2SO_4, filtered and concentrated under reduced pressure to afford the corresponding Boc-protected-Amino Acid-Cyanomethyl ester, which was used without further purification. Note: The cyanomethyl ester is very sensitive to aqueous basic medium.

The Boc-protected-Amino Acid-Cyanomethyl ester (*ca* 0.5 mmol) was treated with neat formic acid (2 mL). The solution was stirred at rt for 12 h before removing all the formic acid under reduced pressure by azeotropic distillation with $CHCl_3$ to afford a pale-yellow oil. The oil was dissolved in minimum amount of THF (*ca.* 2 mL), triturated with excess MTBE or Et_2O until white solid was formed persistently. All the residual solvent was removed under reduced pressure. The white solid was crushed into fine powder, rinsed thoroughly with Et_2O (10 mL) and dried over vacuum for overnight. The typical yield over two steps was 50%. Note: The final product is very sensitive to water and alcohol which cause saponification or transesterification.

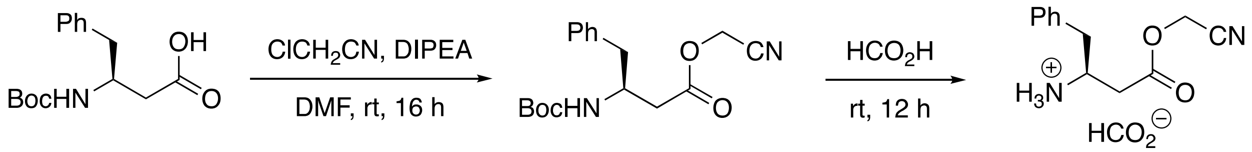

(*S*)-4-(cyanomethoxy)-4-oxo-1-phenylbutan-2-aminium formate

^{1}H NMR (500 MHz, $CDCl_3$) δ 9.22 (s, 3H), 8.37 (s, 1H), 7.41 – 7.26 (m, 5H), 4.72 (s, 2H), 3.89 (s, 1H), 3.23 (dd, J = 14.0, 5.6 Hz, 1H), 3.04 – 2.74 (m, 3H).
^{13}C NMR (126 MHz, $CDCl_3$) δ 169.9, 168.2, 135.4, 129.5, 129.2, 127.7, 114.4, 49.4, 49.1, 39.3, 35.8.
HRMS-ESI: calculated for $[C_{12}H_{15}N_2O_2]^+$ 219.1128, found 219.1150.

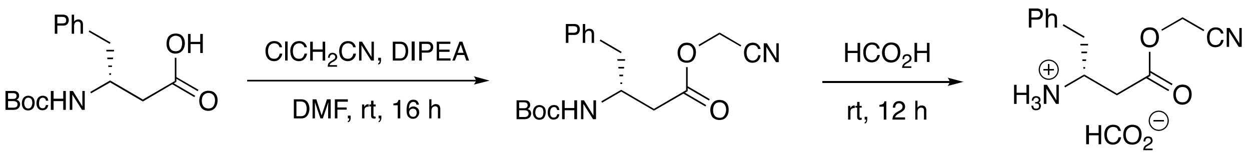

(*R*)-4-(cyanomethoxy)-4-oxo-1-phenylbutan-2-aminium formate

^{1}H NMR (500 MHz, $CDCl_3$) δ 9.15 (s, 3H), 8.36 (s, 1H), 7.43 – 7.20 (m, 5H), 4.70 (s, 2H), 3.87 (s, 1H), 3.22 (dd, *J* = 13.6, 5.6 Hz, 1H), 3.01 – 2.70 (m, 3H).
^{13}C NMR (126 MHz, $CDCl_3$) δ 169.8, 168.3, 135.5, 129.5, 129.2, 127.7, 114.4, 49.3, 49.0, 39.3, 35.9.
HRMS-ESI: calculated for $[C_{12}H_{15}N_2O_2]^+$ 219.1128, found 219.1144.

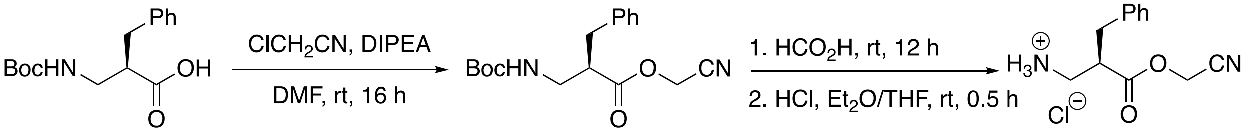

(*R*)-2-benzyl-3-(cyanomethoxy)-3-oxopropan-1-aminium chloride

^{1}H NMR (500 MHz, DMSO-*d*6) δ 8.45 (s, 3H), 7.35 – 7.14 (m, 5H), 4.95 (s, 2H), 3.21 (ddq, *J* = 8.3, 6.0, 3.0 Hz, 1H), 3.08 – 2.96 (m, 2H), 2.90 (dd, *J* = 13.8, 8.0 Hz, 2H).
^{13}C NMR (126 MHz, DMSO-*d*6) δ 171.1, 137.3, 128.9, 128.5, 126.8, 115.6, 49.6, 44.3, 39.1.
HRMS-ESI: calculated for $[C_{12}H_{15}N_2O_2]^+$ 219.1128, found 219.1141.

Procedure for anion metathesis with chloride

After removal of residual formic acid from the Boc deprotection, the oily form product (formate salt) was dissolved in minimum amount of THF (*ca* 2 mL). HCl (1M in Et_2O) (1.5 mL, 3 equiv.) was added. The solution was stirred for 0.5 h before concentration to dryness, and repetitively triturated with excess MTBE or Et_2O until white solid was formed persistently. All the residual solvent was removed under reduced pressure. The white solid was crushed into fine powder, rinsed thoroughly with Et_2O (10 mL) and dried over vacuum for overnight.

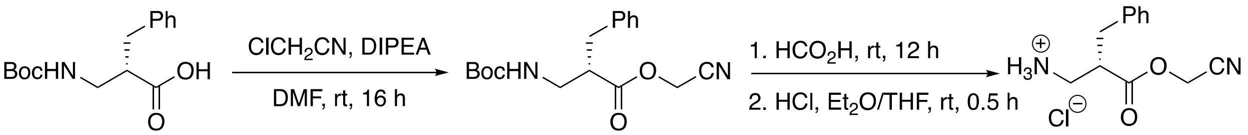

(*S*)-2-benzyl-3-(cyanomethoxy)-3-oxopropan-1-aminium chloride

Following the procedure for anion metathesis with chloride.

^{1}H NMR (500 MHz, DMSO-*d*6) δ 8.37 (s, 3H), 7.32 – 7.20 (m, 5H), 4.95 (s, 2H), 3.19 (td, *J* = 8.2, 4.1 Hz, 1H), 3.06 – 2.87 (m, 4H).
^{13}C NMR (126 MHz, DMSO-*d*6) δ 171.1, 137.3, 128.9, 128.5, 126.8, 115.7, 49.6, 44.4, 39.1.
HRMS-ESI: calculated for $[C_{12}H_{15}N_2O_2]^+$ 219.1128, found 219.1137.

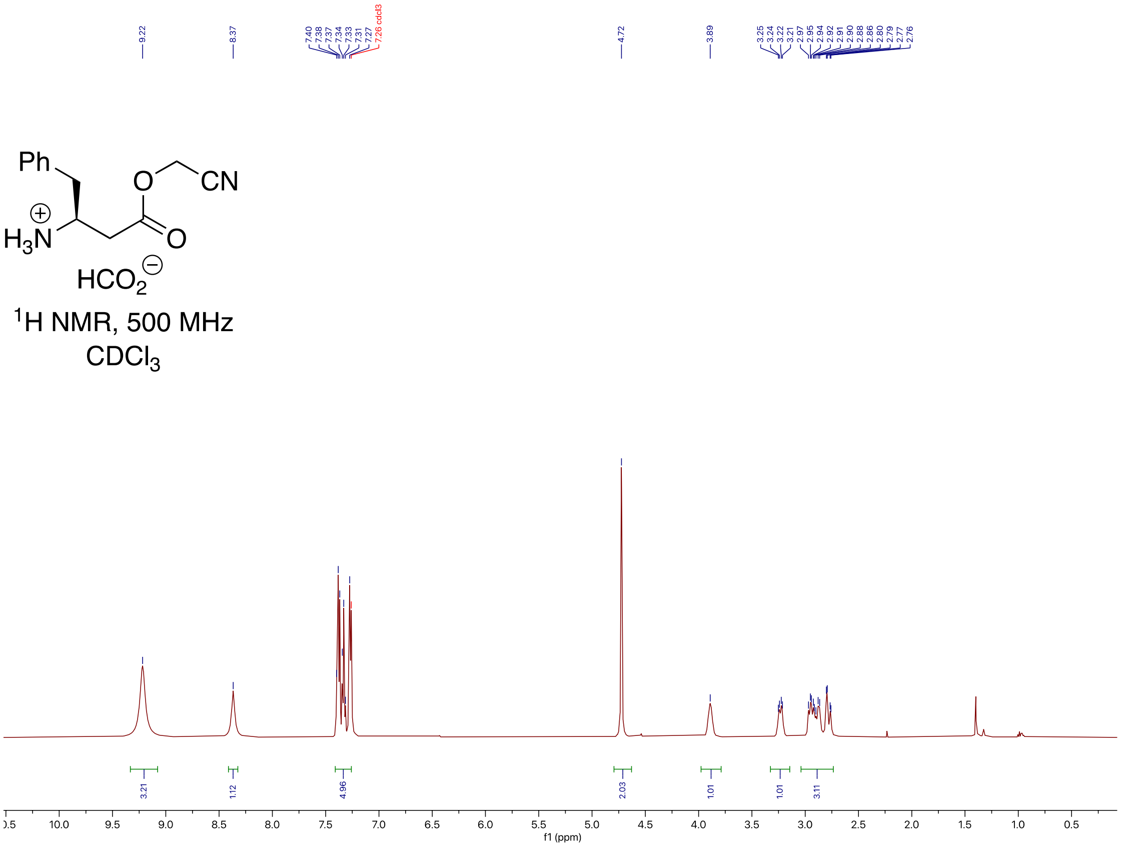

^{1}H NMR, 500 MHz
$CDCl_3$

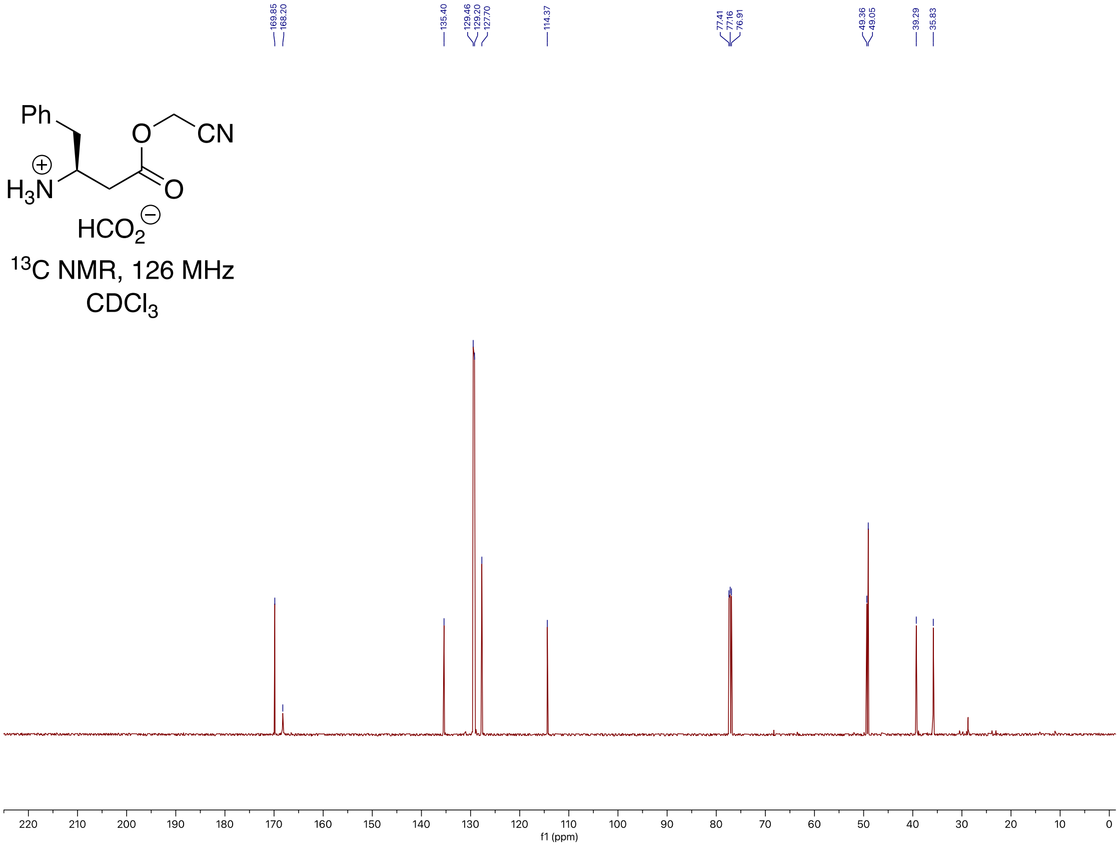

^{13}C NMR, 126 MHz
$CDCl_3$

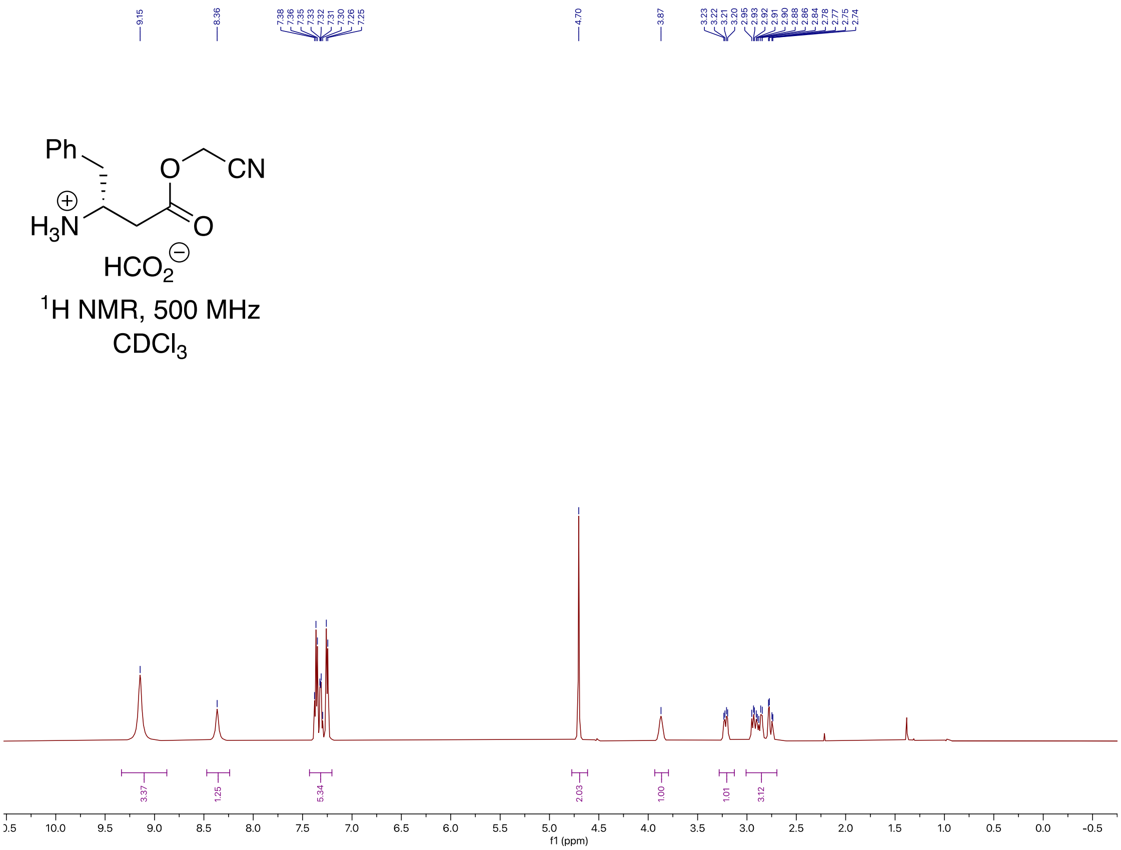
9.15
8.36
7.38 7.36 7.35 7.33 7.32 7.31 7.30 7.26 7.25
4.70
3.87
3.23 3.22 3.21 3.20 2.95 2.93 2.92 2.91 2.90 2.88 2.86 2.84 2.78 2.77 2.75 2.74
Ph
O
CN
H3N
O
HCO2
1H NMR, 500 MHz
CDCl3
3.37
1.25
5.34
2.03
1.00
1.01
3.12
f1 (ppm)

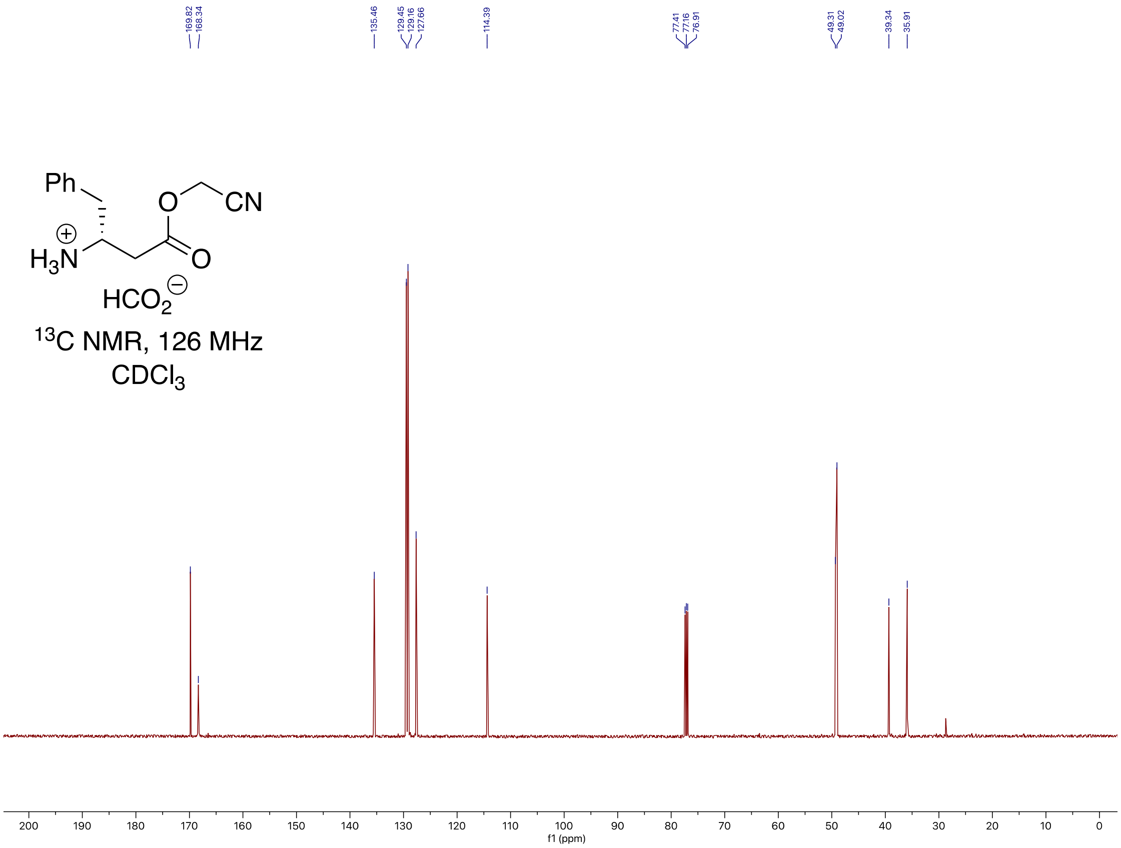
169.82
168.34
135.46
129.45 129.16 127.66
114.39
77.41 77.16 76.91
49.31 49.02
39.34
35.91
Ph
O
CN
H3N
O
HCO2
13C NMR, 126 MHz
CDCl3
f1 (ppm)

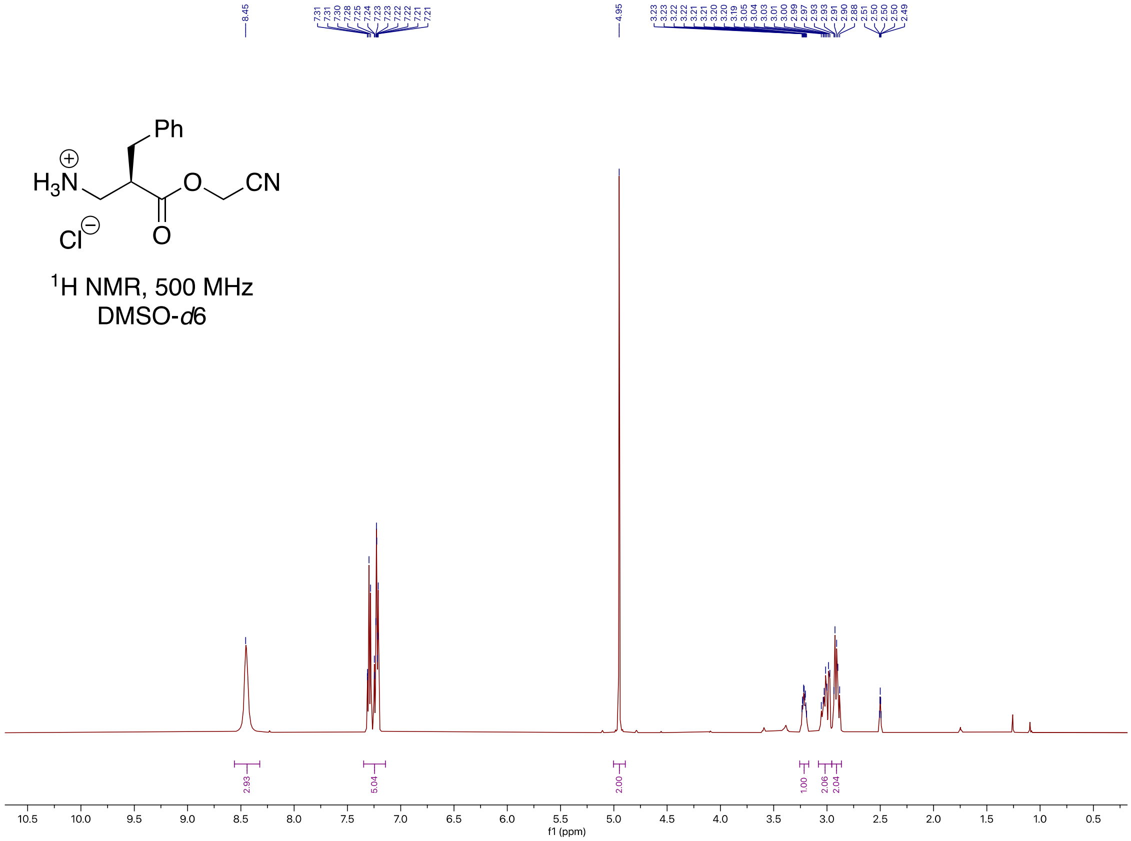
Ph
H3N
CN
Cl
1H NMR, 500 MHz
DMSO-d6
f1 (ppm)

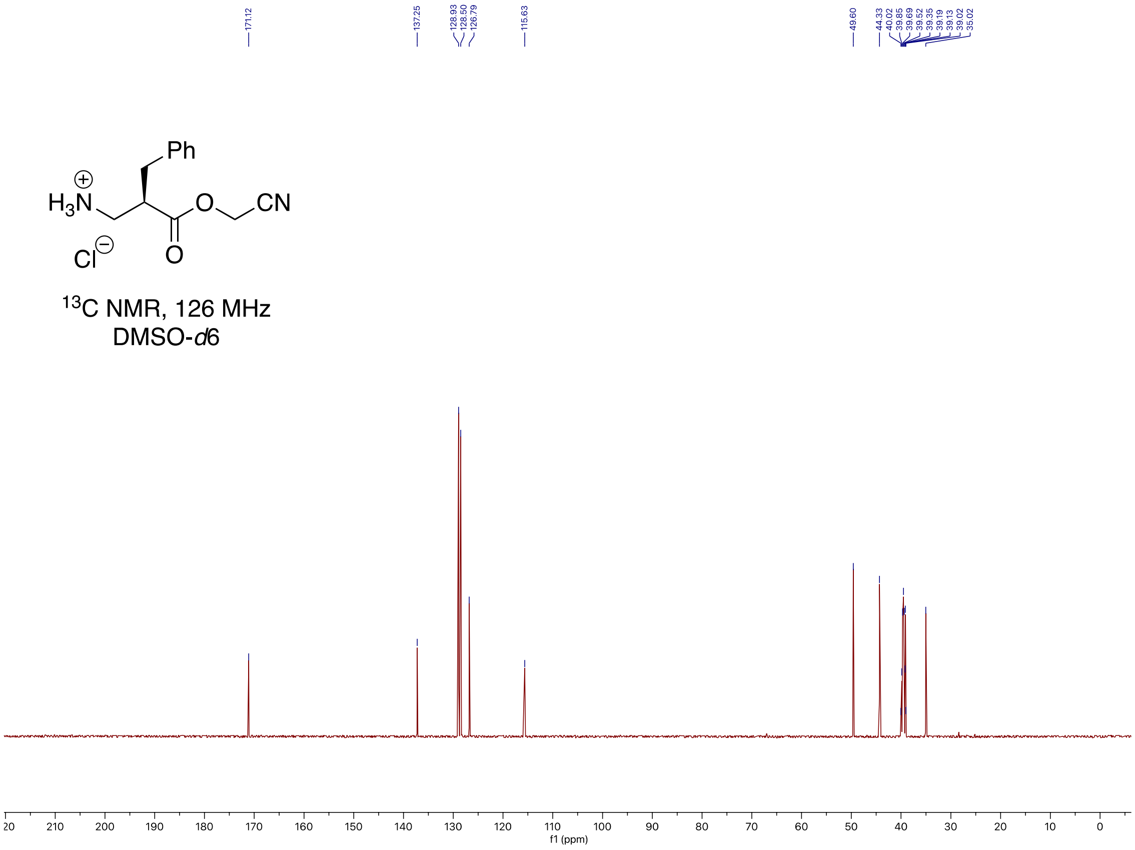
Ph
H3N
CN
Cl
13C NMR, 126 MHz
DMSO-d6
f1 (ppm)

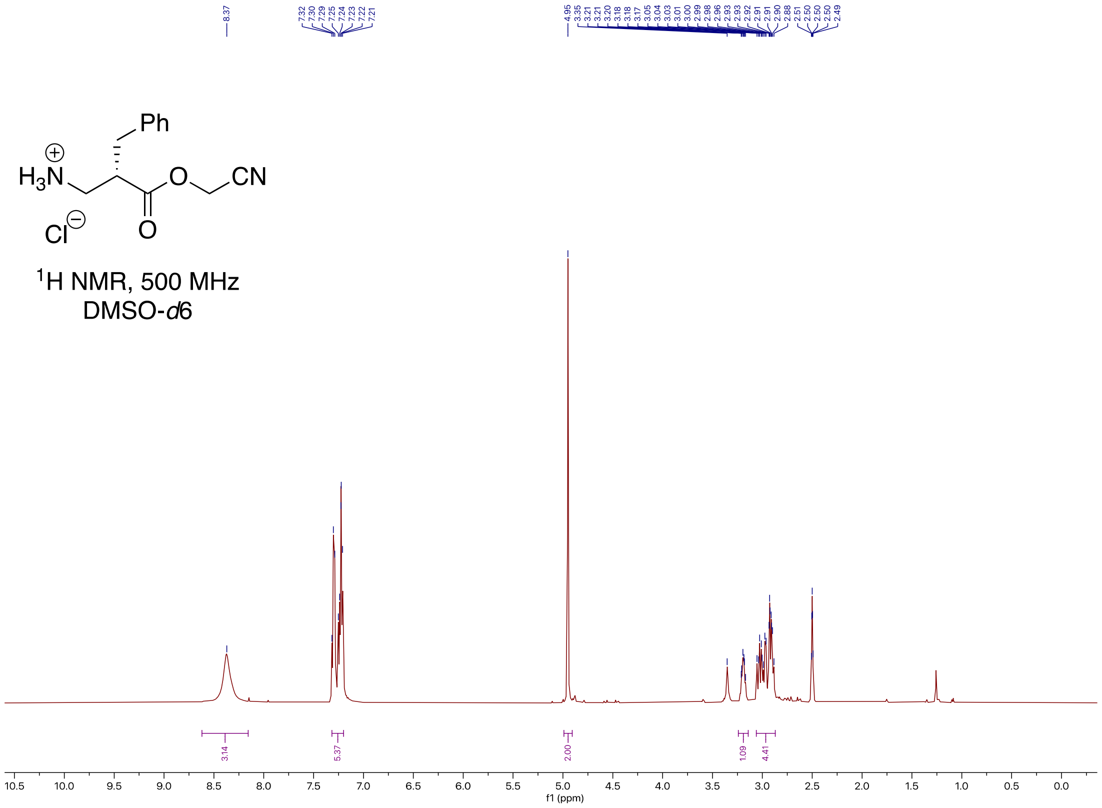

Ph
$H_3N^{\oplus}$
O
CN
$Cl^{\ominus}$
O
^{1}H NMR, 500 MHz
DMSO-*d*6
f1 (ppm)

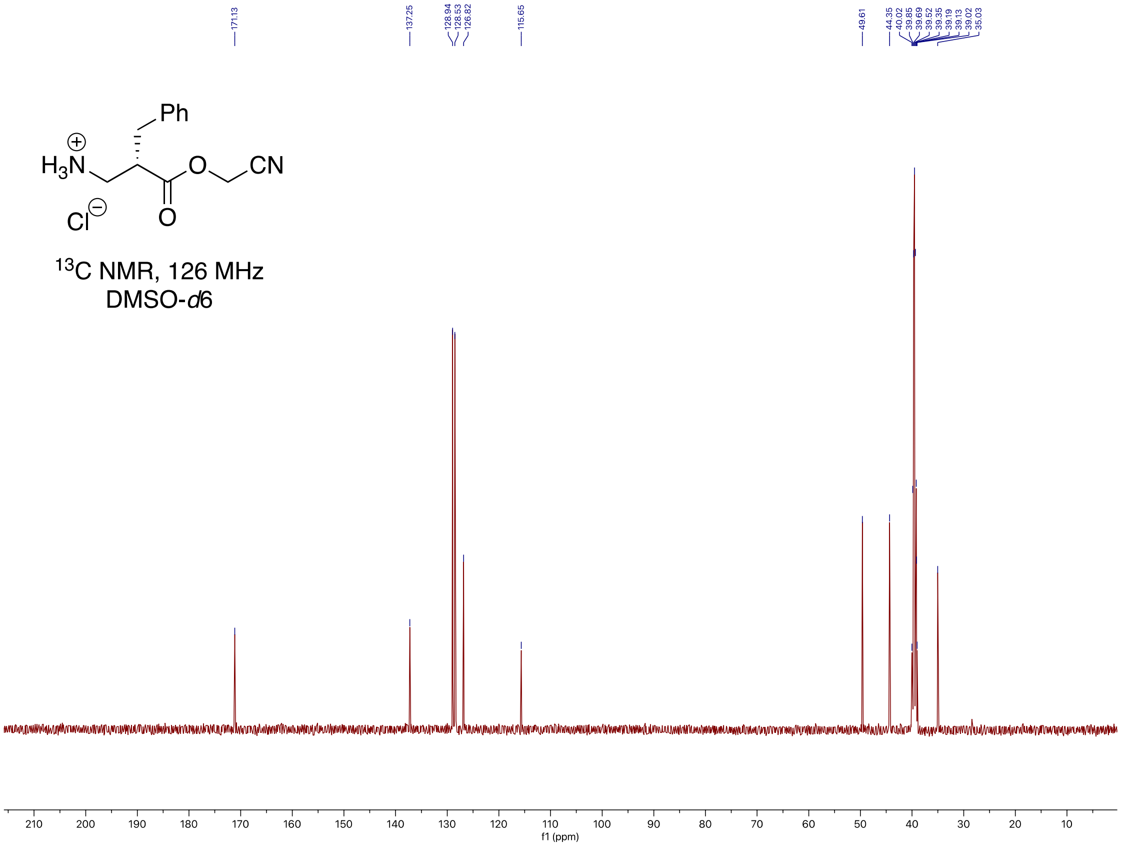

Ph
$H_3N^{\oplus}$
O
CN
$Cl^{\ominus}$
O
^{13}C NMR, 126 MHz
DMSO-*d*6
f1 (ppm)

Synthesis of α-amino acid-cyanomethyl esters

To a 5-mL round-bottom flask, N-Boc protected amino acid (0.5 mmol) was dissolved in 1 mL of tetrahydrofuran. Flask was then charged with 315 μL of chloroacetonitrile (5.0 mmol, 10 eq.), followed by addition of 100 L of N,N-diisopropylethylamine (0.6 mmol, 1.2 eq.). Flask was capped with septa and stirred at room temperature overnight, 16 hours. Solvent was removed via rotary evaporation then the crude material was purified by reverse-phase flash chromatography, 0-100% acetonitrile in water, holding at 60% acetonitrile until product was collected. Solvent removed via rotary evaporation, where the resulting oil was dissolved in 1 mL of tetrahydrofuran for deprotection. To the resulting solution, 1.9 mL of trifluoroacetic acid (25 mmol, 50 eq.) was added, and allowed to stir at room temperature for 2 hours. Upon completion, the solvent was removed followed by purification by reverse-phase flash chromatography utilizing a 2% acetonitrile in water mobile phase. Solvent was removed by lyophilization to yield target materials as colorless oils at 54% and 49% yield for phenylalanine and 4-azidophenylalanine derivatives respectively. Note: The final product is very sensitive to water and alcohol which cause saponification or transesterification.

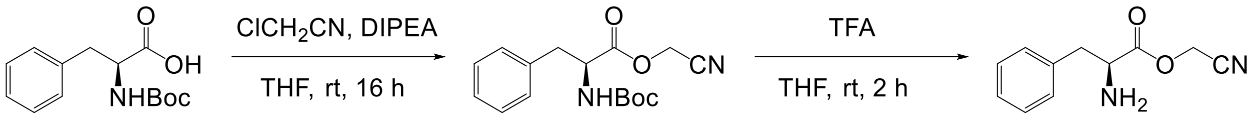

Cyanomethyl-ester-L-phenylalanine

^{1}H NMR (400 MHz, DMSO-*d*6) δ 8.58 (s, 2H), 7.33 (m, 5H), 5.10 (d, 2H), 4.47 (t, 1H), 3.12 (m, 2H). ESI-MS: calculated for $[C_{11}H_{13}N_2O_2]^+$ = 205.10, found 205.24.

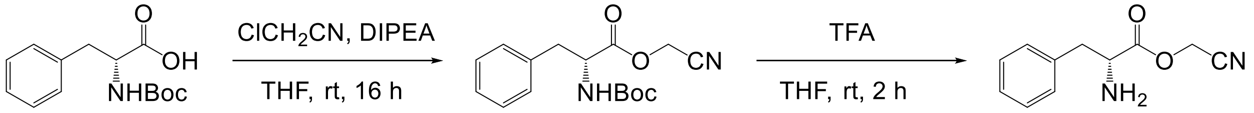

Cyanomethyl-ester-D-phenylalanine

^{1}H NMR (400 MHz, DMSO-*d*6) δ 8.71 (s, 2H), 7.32 (m, 5H), 5.08 (d, 2H), 4.45 (t, 1H), 3.12 (m, 2H). ESI-MS: calculated for $[C_{11}H_{13}N_2O_2]^+$ = 205.10, found 205.16.

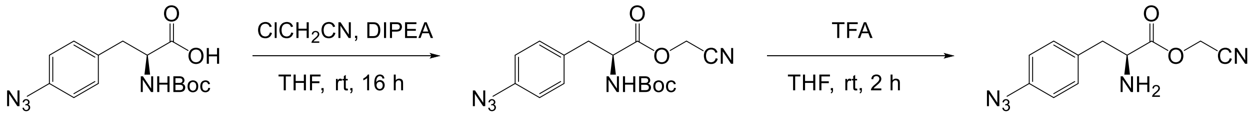

4-Azido-Cyanomethyl-Ester-L-Phenylalanine

^{1}H NMR (400 MHz, DMSO-*d*6) δ 8.45 (s, 2H), 7.29 (d, 2H), 7.09 (d, 2H), 5.09 (d, 2H), 4.43 (t, 1H), 3.11 (m, 2H). ESI-MS: calculated for $[C_{11}H_{12}N_5O_2]^+$ = 246.10, found 246.15.
